# Supplementary material for: Reference-based chemical-genetic interaction profiling to elucidate small molecule mechanism of action in Mycobacterium tuberculosis
Source: Nat Commun. 2025 Nov 3;16:9673. doi: 10.1038/s41467-025-64662-x (PMC12583738; doi:10.1038/s41467-025-64662-x)
Supplement: Supplementary file 1 — Supplementary Information [file 41467_2025_64662_MOESM1_ESM.pdf]

Supplementary Information

Reference-based chemical-genetic interaction profiling to elucidate small molecule mechanism of action in *Mycobacterium tuberculosis*

Austin N. Bond[1#], Marek Orzechowski[1#], Shuting Zhang[1#], Ishay Ben-Zion[1], Allison Lemmer[1], Nathaniel Garry[1], Katie Lee[1], Michael Chen[1], Kayla Delano[1], Emily Gath[1], A. Lorelei Golas[1], Raymond Nietupski[1], Michael Fitzgerald[1], Sabine Ehrt[2], Eric J. Rubin[3], Christopher M. Sassetti[4], Dirk Schnappinger[2], Noam Shoresh[1], Diana K. Hunt[1], James E. Gomez[1]*, Deborah T. Hung[1,5,6,7]*

[1]Broad Institute of MIT and Harvard; Cambridge, Massachusetts, 02142; USA

[2]Department of Microbiology and Immunology, Weill Cornell Medical College, New York, New York; USA

[3]Department of Immunology and Infectious Diseases, Harvard T. H. Chan School of Public Health, Boston, MA; USA

[4]Department of Microbiology, UMass Chan Medical School. Worcester, MA; USA

[5]Department of Molecular Biology and Center for Computational and Integrative Biology, Massachusetts General Hospital; Boston, Massachusetts, 02114; USA

[6]Department of Genetics, Harvard Medical School; Boston, Massachusetts, 02115; USA

[7]Center for Integrated Solutions in Infectious Diseases, Broad Institute of Harvard and MIT, Cambridge, MA; USA

[#] These authors contributed equally to this work

[*]Corresponding authors:

James E. Gomez, jgomez@broadinstitute.org, Deborah T. Hung, hung@molbio.mgh.harvard.edu

Table of Contents

Supplementary Figures 2
Supplementary Tables 21
Supplementary Notes 26

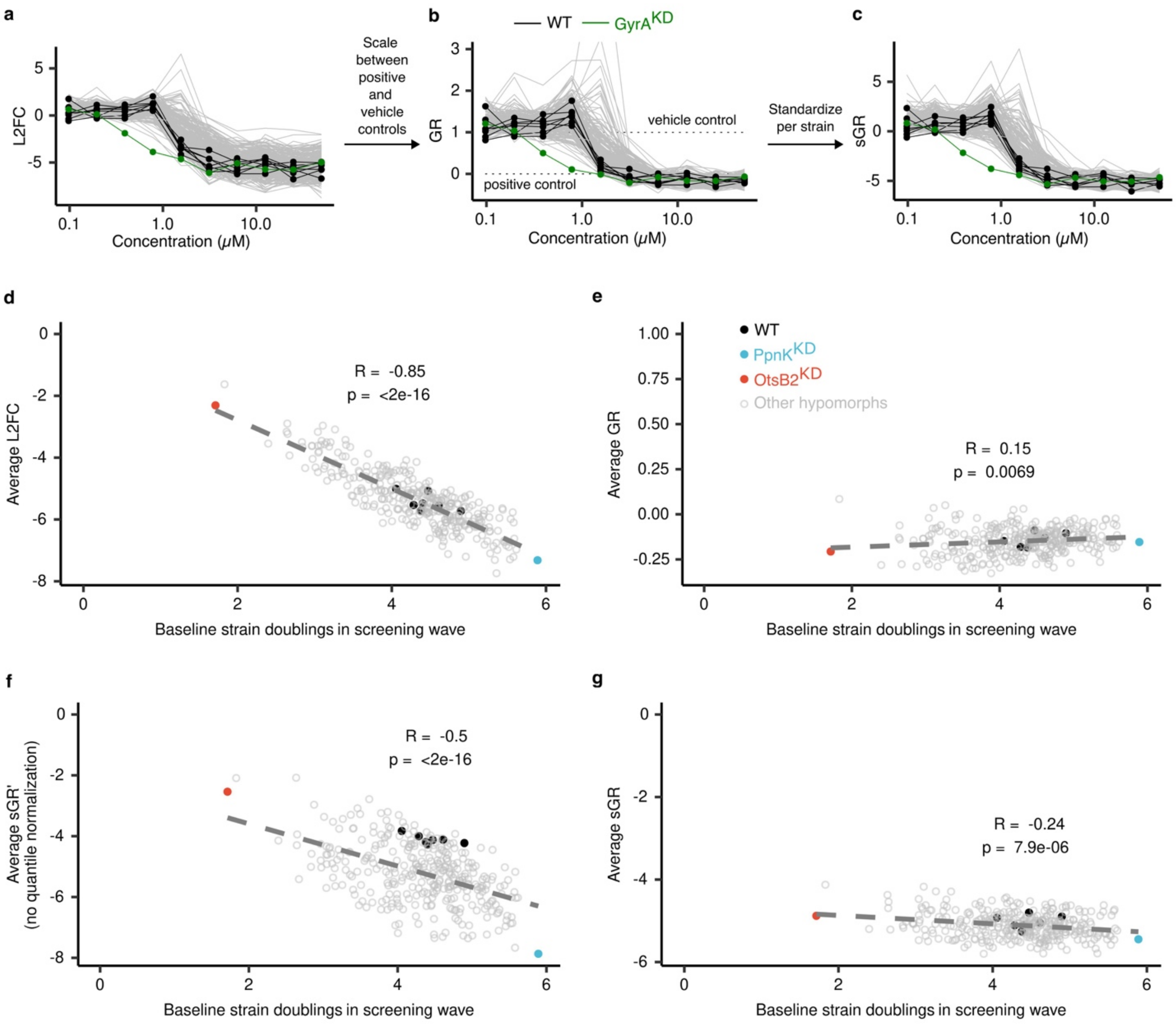

Supplementary Fig. 1 | Growth scores and strain normalization to reduce dependence on baseline strain growth rates. a-c, Example growth scores (L2FC, GR, sGR) across different concentrations of the fluoroquinolone nadifloxacin for 7 wild-type H37Rv barcoded strains (black), GyrA hypomorph (green), and 332 other hypomorphs in the pool (gray). Dashed lines represent vehicle (DMSO, GR = 1) and positive (498 nM rifampin, GR = 0) controls used for scaling. For **b** only, the maximum y-axis shown is limited to 3. **d-g,** Growth scores L2FC, GR, sGR' (= sGR with no strain GR quantile normalization step), and sGR for each strain were averaged over the three highest, fully inhibitory doses of nadifloxacin shown in **a-c** and correlated to each strains' average baseline doublings in the specific screening wave. Black circles are wild-type H37Rv barcoded strains, red circle is the slowest growing strain on average in the screening wave OtsB2, blue circle is the fastest growing strain on average in the screening wave PpnK, and gray open circles are all other hypomorphs. Dark grey dashed line is the best-fit linear regression line and Pearson correlation coefficient and p-value shown. **d,** L2FC is strongly, inversely correlated to strains' baseline doublings, while in **e** GR shows weak-to-no association as expected for doses of compound that completely and equally inhibit the strain pool. **f**, Robust z-scoring reintroduces the inverse correlation (in sGR'), but quantile normalizing strain GR distributions prior to z-scoring, in **g** (sGR), reduces the association. For more detail on mean-variance dependence in GR based on baseline growth and plots for

additional example reference set compounds see Supplementary Fig. 3. L2FC, log2-fold change; GR, compound-induced growth rate; sGR, standardized GR. Source data are provided as a Source Data file.

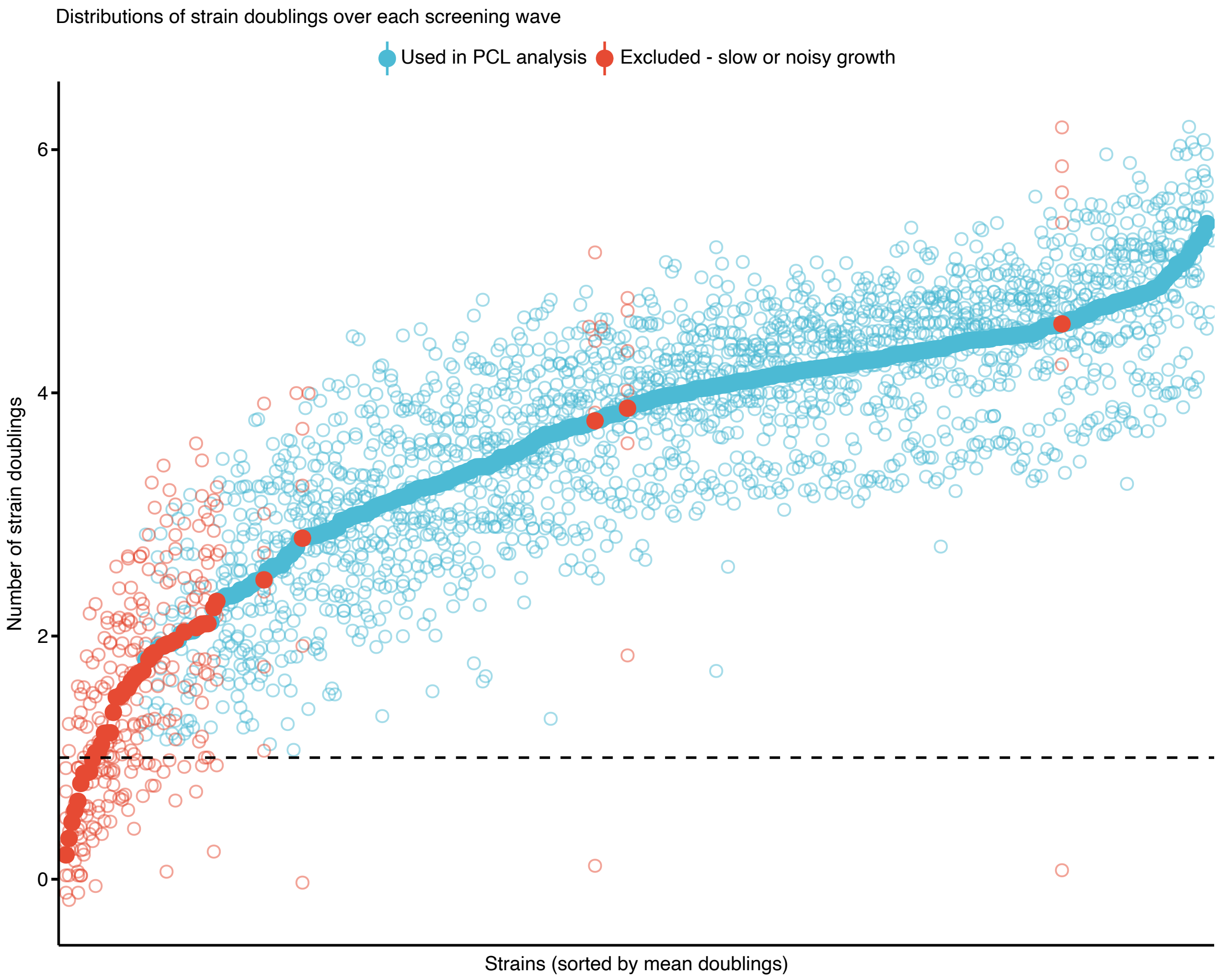

Supplementary Fig. 2 | Variation in strain doublings/baseline growth rates. Dot plot of each strain's baseline doublings (open circle) in the six screening waves and the average across all six waves (filled circle). Black dashed line represents a 1 doubling cutoff for slow growth. Data for 387 strains are shown: 340 (blue) that passed quality control and were used in the PCL analysis (i.e., grew at least 1 doubling in all six screening waves and no conditions with GR > 20) and 47 (red) that failed quality control and were excluded (i.e., 42 for slow growth, <1 doubling in at least one screening wave and 5 for noisy growth, conditions with GR > 20). For underlying data and strain identity, including 84 other strains that were present in at least one but not all screening waves and were also excluded from the combined PCL analysis (Supplementary Note 2), see Supplementary Data 2 and provided Source Data file. PCL, Perturbagen Class; GR, compound-induced growth rate.

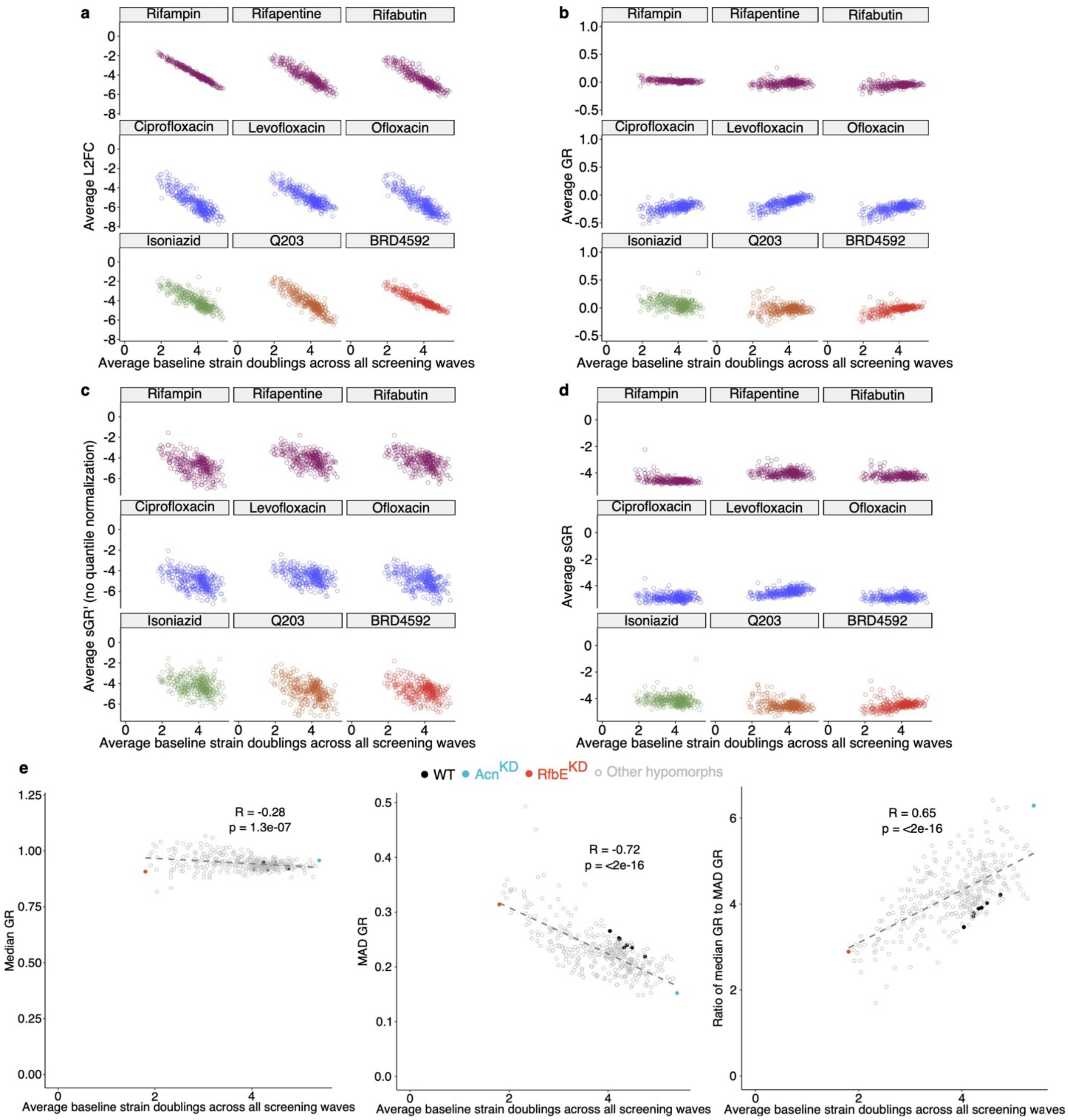

Supplementary Fig. 3 | Mean-variance dependence in GR based on baseline growth and addressing dependence for hit-calling through quantile normalization. As in Supplementary Fig. 1d-g, growth scores L2FC, GR, sGR' (= sGR with no strain GR quantile normalization step), and sGR for each strain were averaged over fully inhibitory, >4X MIC doses of 9 diverse, exemplary reference set compounds (67 total compound-doses) and correlated to each strains' average doublings (L2FC of onboard, 256 nM or 498 nM rifampin) across the screening waves. Average growth scores for each strain were calculated and plotted separately for each compound (rifamycins in purple, fluoroquinolones in blue, isoniazid in green, Q203 in orange, and BRD4592 in red). **a,** L2FC is strongly, inversely correlated to strains' base doublings while in **b** GR shows weak-to-no association, as expected for doses of compound that completely and equally inhibit

the strain pool. **c**, Robust z-scoring reintroduces the inverse correlation (in sGR'), but quantile normalizing strain GR distributions prior to z-scoring, in **d** (sGR), reduces the association. For reference set compounds and doses considered fully inhibitory in **a-d** see Supplementary Note 3. **e**, Scatter plots of each strains' average doublings versus their (**left**) median GR, (**middle)** median absolute deviation (MAD) in GR, and (**right)** ratio of median GR to MAD GR over all screened conditions. Black circles are wild-type H37Rv barcoded strains, red circle is the slowest growing strain on average across all screening waves RfbE, blue circle is the fastest growing strain on average across all screening waves Acn, and gray open circles are all other hypomorphs. Dark gray dashed line is the best-fit linear regression line and Pearson correlation coefficient and p-value shown. L2FC, log2-fold change; GR, compound-induced growth rate; sGR, standardized GR. Source data are provided as a Source Data file.

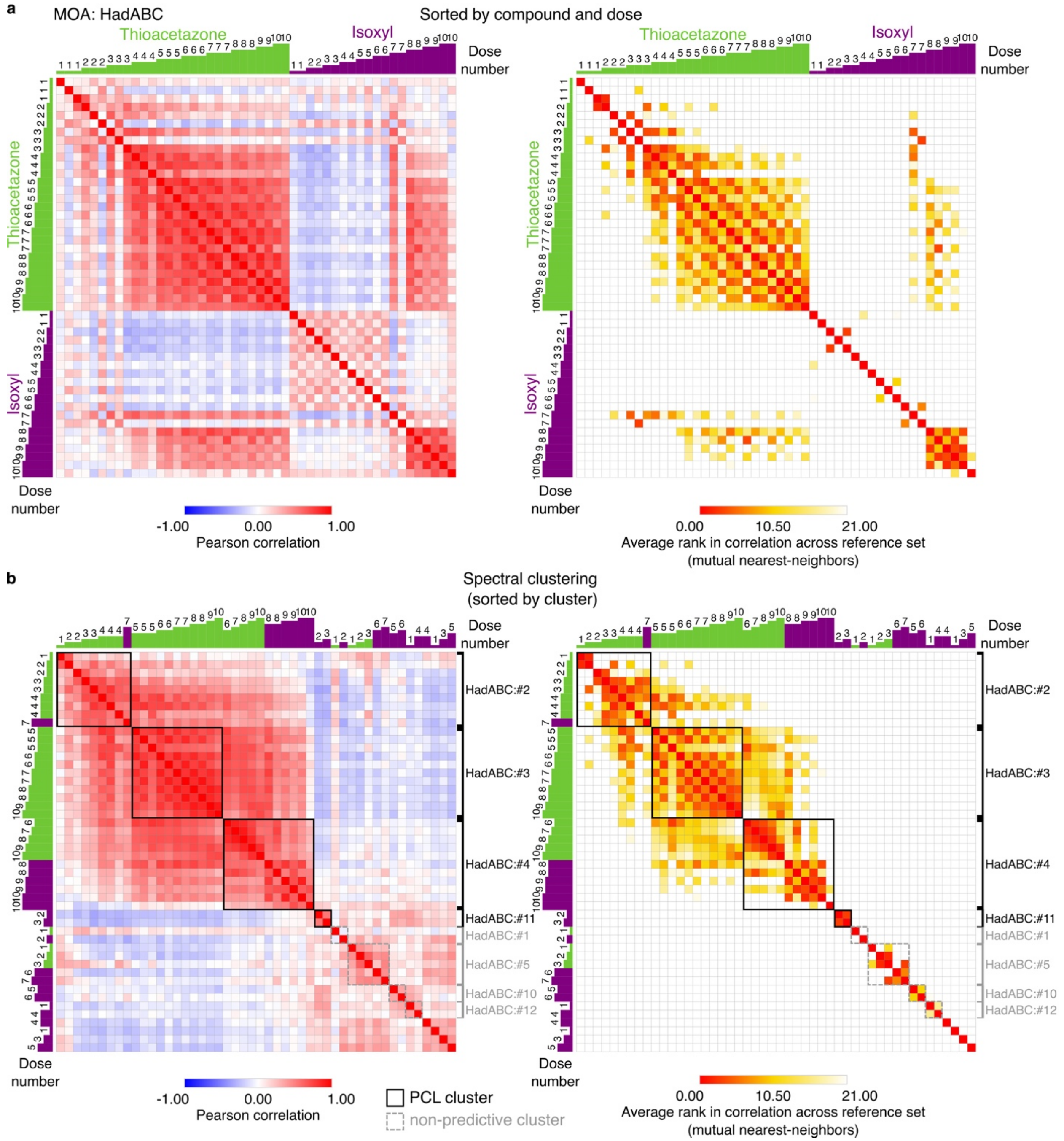

Supplementary Fig. 4 | Spectral clustering results for example MOA HadABC. a, Heatmaps of (**left**) Pearson correlation between CGI profiles and (**right**) average rank in correlation across all reference set CGI profiles for the two compounds, thioacetazone (green) and isoxyl (purple), with annotated MOA HadABC in mycolic acid synthesis. Thioacetazone was screened in dose-response in three of the six screening waves and isoxyl was screened in dose-response in two. Dose number (1 = 0.1 µM to 10 = 50 µM in two-fold dilution series) is depicted in green/purple bar plots outside the heatmaps. Heatmaps in **a** are sorted by compound and dose from low-to-high concentration. A threshold of ≤ 20 was applied to the (**right**) average rank of correlation matrix to connect HadABC CGI profiles that were mutual nearest-neighbors and create an adjacency matrix (1 = connected conditions, 0 = disconnected conditions) as input

for spectral clustering. For more detail on the steps of spectral clustering, see Supplementary Note 7. **b**, as in **a** but heatmaps re-sorted by cluster membership from spectral clustering. PCL clusters and non-predictive clusters are outlined in black and dashed-gray squares respectively, while singleton CGI profiles that did not cluster with others are unlabeled. For HadABC:#4, its PCL similarity score distribution across all reference set CGI profiles and determination of its high-confidence similarity score threshold is shown in Supplementary Fig. 8a. Heatmaps were performed in Morpheus (https://software.broadinstitute.org/morpheus). CGI, chemical-genetic interaction; MOA, mechanism of action; PCL, Perturbagen Class. Source data are provided as a Source Data file.

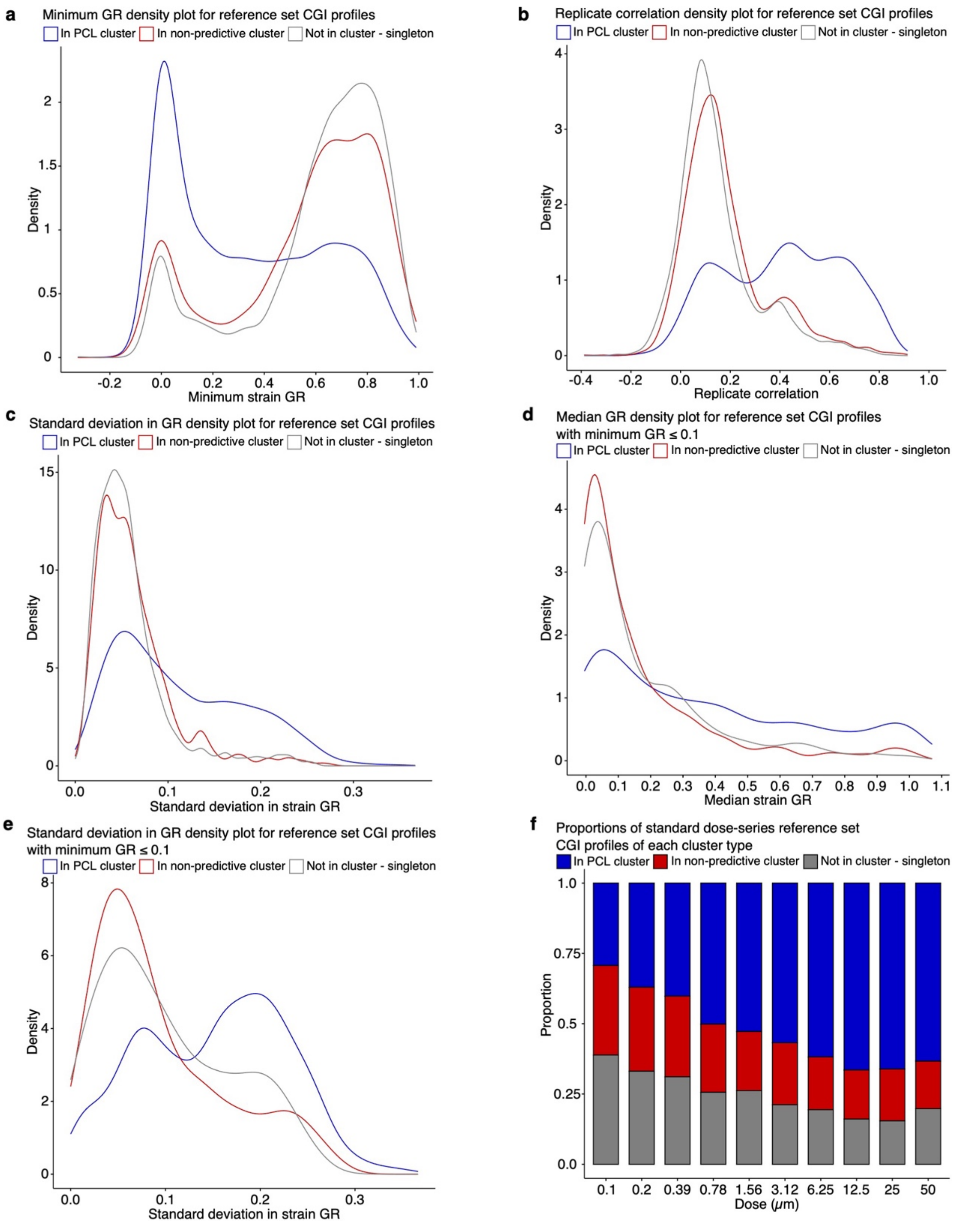

Supplementary Fig. 5 | Distributions of strain inhibition, replicate correlation, and dose for reference set CGI profiles based on their cluster membership from spectral clustering. Of 9,427 chemical-genetic interaction (CGI) profiles in the reference set, 55% (5,134) were members of a Perturbagen Class (PCL) cluster (blue), 22% (2,095) were members of a non-predictive cluster (red), and 23% (2,198) were singletons (not in a cluster; gray) from spectral clustering for each mechanism of action (MOA) (Supplementary Data 3). **a**, Density plot of minimum strain compound-induced growth rate (GR)

(maximum strain inhibition) measured for each condition; CGI profiles in PCLs had greater activity with a large proportion showing nearly complete inhibition of at least one strain (minimum strain GR ≤ 0.1). **b** and **c,** Density plots of condition replicate correlation (*n* = 2 biologically independent experiments) and standard deviation in strain GR for each reference set CGI profile; conditions that were not in PCLs had poorer replicate reproducibility and lesser variation in strain GR likely due to lack of real, reproducible biological signal (inhibition) and instead strain shuffling due to random noise. **d** and **e**, Density plots of median strain GR and standard deviation in strain GR for the 2,263 reference set conditions with nearly complete inhibition of at least one strain (minimum strain GR ≤ 0.1) (78% in PCLs, 13% in non-predictive clusters and 9% singletons); active conditions that were not in PCLs killed most or all strains entirely (median strain GR ≈ 0 and low variance in strain GR) while those in PCLs exhibited wider variation in strain GR capturing meaningful, biological signal for discriminating between MOAs. All 437 reference set compounds were screened twice in two independent screening waves across a standard 10-point, two-fold dilution series comprising 8,740 standard dose-series CGI profiles. **f**, Proportions of standard dose-series CGI profiles that were members of a PCL cluster, non-predictive cluster, or not in a cluster. Underlying GR scores shown were strain curve-fit GR values, as described in Supplementary Note 6, which reduced dose-to-dose noise; however, using raw GR values did not significantly change the overall observations. Source data are provided as a Source Data file.

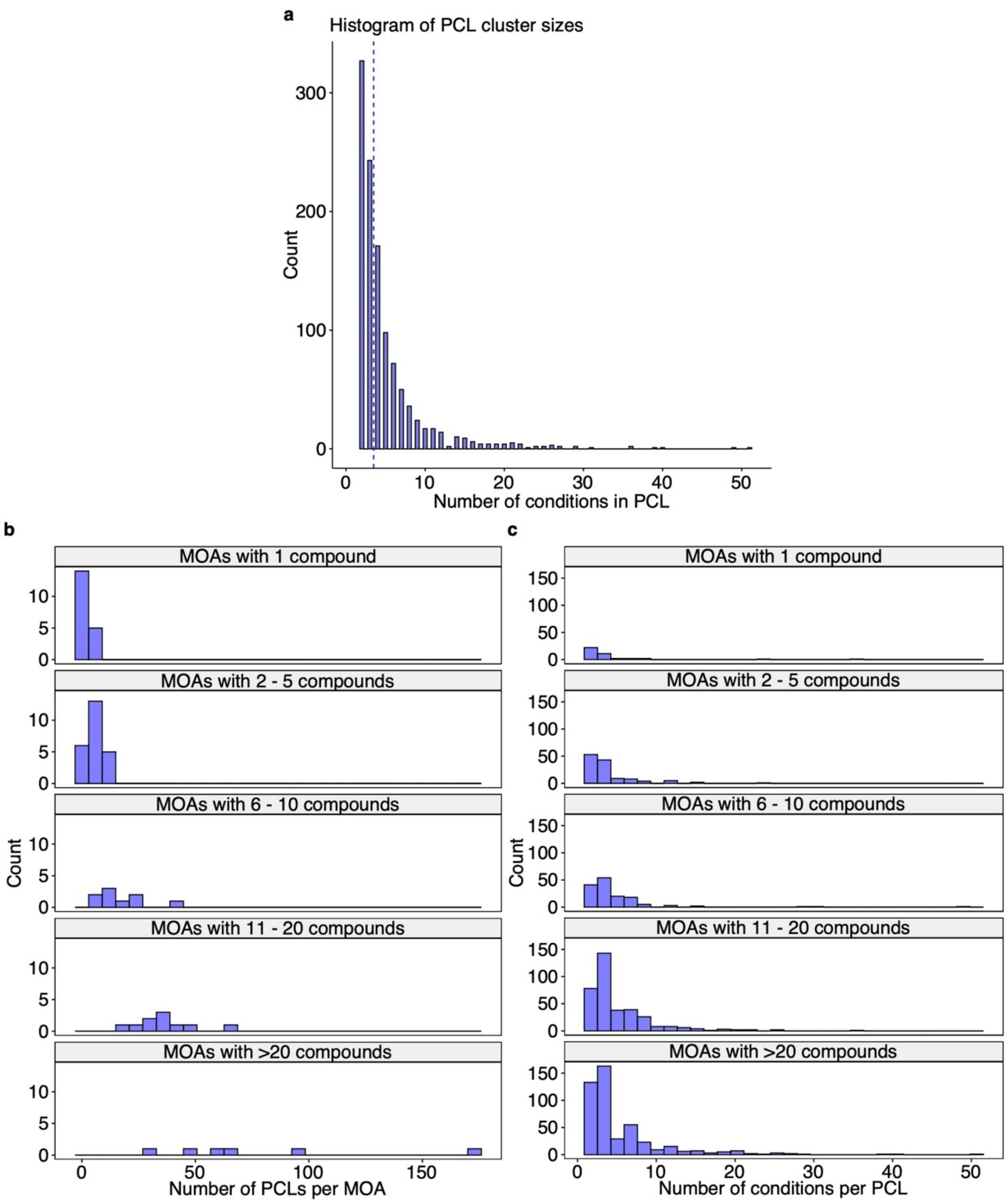

Supplementary Fig. 6 | Distribution of PCL cluster sizes. a, Histogram of the number of reference set conditions (CGI profiles) in each of the 1,140 PCL clusters. Blue dashed line indicates the median PCL cluster size of 3.5. **b**, Histograms of the number of PCL clusters constructed per reference MOA for each of the 68 MOAs, grouped by MOA size (i.e., number of reference compounds in the annotated MOA). The number of PCL clusters found via spectral clustering for a given MOA was generally associated with its size. **c**, as in **a**, but histograms grouped by MOA size across the 68 MOAs. Regardless of MOA size, the number of conditions in a PCL cluster varied widely while larger MOAs showed greater enrichment for large PCLs. In both **b** and **c**, 3 single-compound MOAs are excluded for which no PCL clusters were found. CGI, chemical-genetic interaction; PCL, Perturbagen Class; MOA, mechanism of action. Source data are provided as a Source Data file.

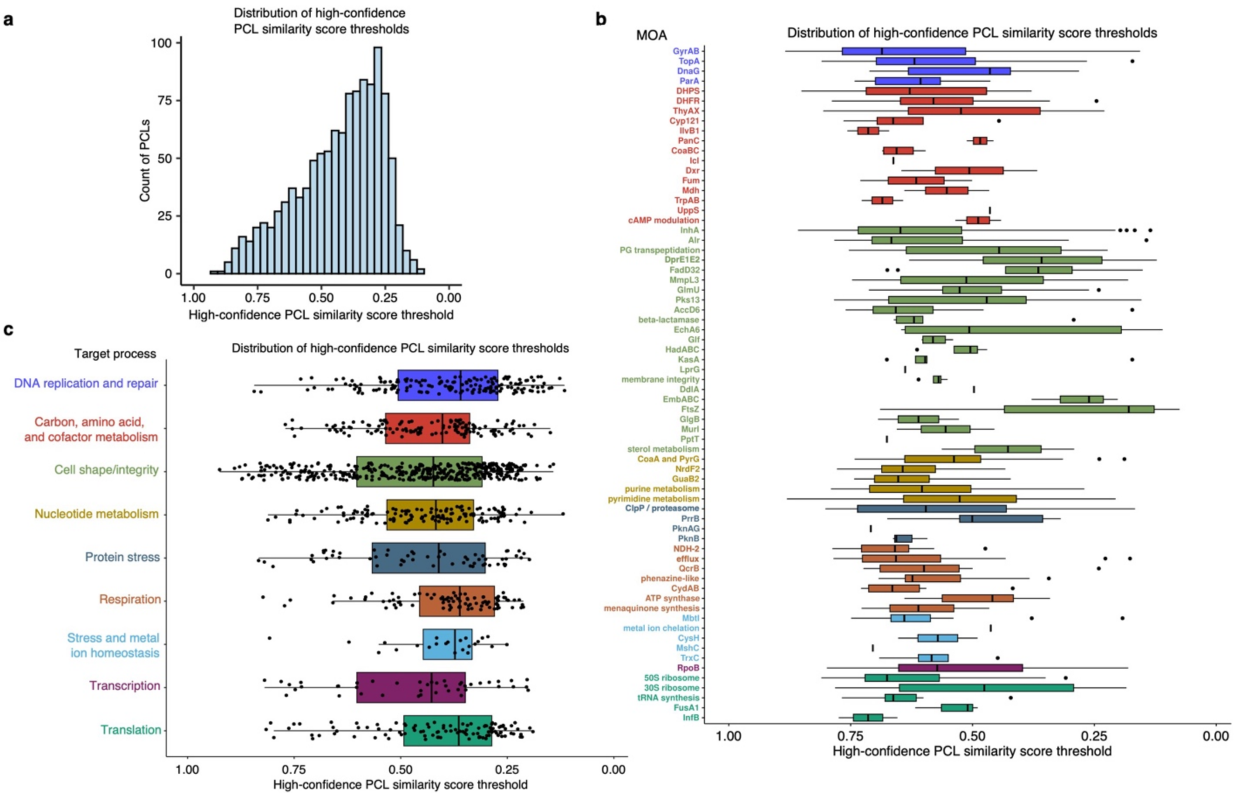

Supplementary Fig. 7 | Distribution of high-confidence PCL similarity score thresholds. a, Histogram of the high-confidence PCL similarity score thresholds defined for each of the 1,140 PCL clusters, where PCL similarity score is the median of the Pearson correlation coefficients between a test CGI profile and all CGI profiles in a PCL (excluding self-similarity if the test CGI profile is in the PCL). As depicted in Fig. 2c, each PCL's high-confidence threshold was defined as the PCL similarity score of the furthest (least similar) in-MOA, reference set CGI profile before the closest (most similar) out-of-MOA, reference set CGI profile. This corresponds to the minimum PCL similarity score where PCL confidence score = 1 (for examples, see Supplementary Fig. 8). PCLs showed a wide range of high-confidence thresholds, owing to the diversity of MOAs represented in the reference set and the different strengths and patterns of strain sensitivities that they elicited (see Fig. 3c and Supplementary Fig. 11b). Consequently, considering high similarity alone, and not each PCL's unique distribution of PCL similarity scores, when making MOA predictions for reference set compounds performed worse in LOOCV (Supplementary Table 1). **b**, As in **a** but boxplot of the high-confidence PCL similarity score thresholds for each PCL cluster grouped and colored by MOA. **c**, As in **a** but jittered boxplot of the high-confidence PCL similarity score thresholds for each PCL cluster grouped and colored by high-level biological target process as in Fig. 1a and Supplementary Data 1. PCL, Perturbagen Class; CGI, chemical-genetic interaction; MOA, mechanism of action; LOOCV, leave-one-out cross-validation; in-MOA, sharing the same MOA as the PCL conditions; out-of-MOA, distinct MOA from the PCL conditions. Source data are provided as a Source Data file.

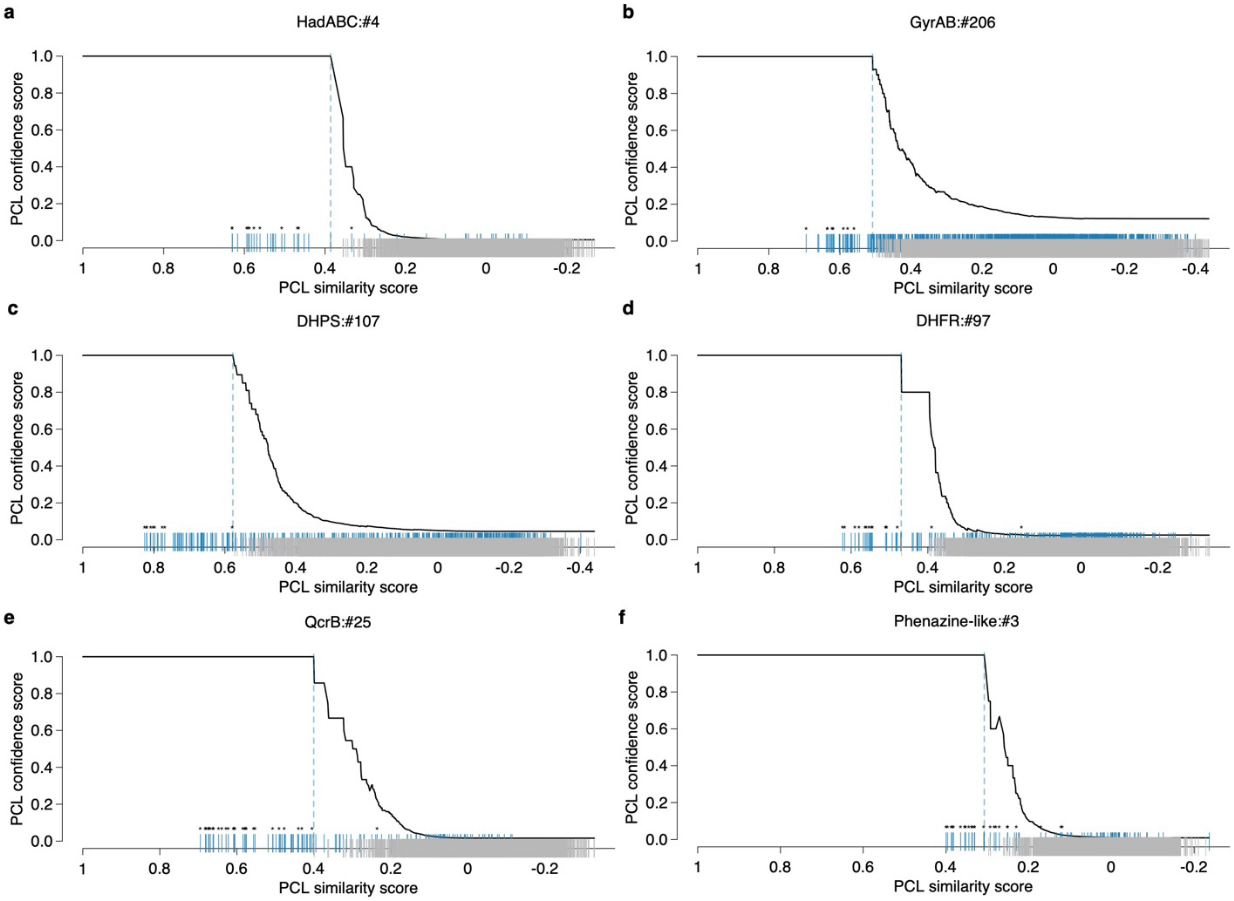

Supplementary Fig. 8 | High-confidence similarity score thresholds and mappings of PCL similarity score to PCL confidence score for example PCLs. For 6 representative PCLs, a rug plot of the distribution of similarity scores to a given PCL across all reference set CGI profiles. Overlayed (black line) is the mapping of PCL similarity score to PCL confidence score. As in Fig. 2c, blue ticks indicate in-MOA reference set CGI profiles while gray ticks indicate out-of-MOA reference set CGI profiles. Asterisks indicate that the CGI profile is a member of the given PCL cluster. As depicted in Fig. 2c, the dashed, light blue line indicates the PCL's defined high-confidence similarity score threshold, i.e., the similarity score of the furthest in-MOA CGI profile before the first out-of-MOA CGI profile. For each observed PCL similarity score X_i, PCL confidence score is calculated as the fraction of unique reference set compounds with similarity score $X \geq X_i$ that are in-MOA. For a CGI profile from an unknown compound, its confidence score to a particular PCL is estimated by fitting its similarity score to the mapping found empirically using the reference set CGI profiles via linear interpolation and estimates the likelihood of (or proportion of evidence supporting) the unknown compound sharing the PCL's MOA. **a**, PCL of mycolic acid synthesis inhibitors: HadABC:#4, composed of 11 CGI profiles representing 2 unique compounds. **b**, PCL of gyrase inhibitors: GyrAB:#206, composed of 8 CGI profiles representing 8 unique compounds. **c-d**, PCLs of folate biosynthesis inhibitors: DHPS:#107 composed of 9 CGI profiles representing 8 unique compounds, and DHFR:#97 composed of 15 CGI profiles representing 3 unique compounds. **e-f**, PCLs of respiration inhibitors: QcrB:#25 composed of 29 CGI profiles representing 4 unique compounds, and Phenazine-like:#3 composed of 24 CGI profiles representing 4 unique compounds. Strain sensitization of PCLs in **e-f** are shown in Fig. 3c. For HadABC:#4, Pearson correlation between HadABC CGI profiles and their average rank of correlation across all

reference set CGI profiles is shown in Supplementary Fig. 4b. PCL, Perturbagen Class; MOA, mechanism of action; CGI, chemical-genetic interaction. Source data are provided as a Source Data file.

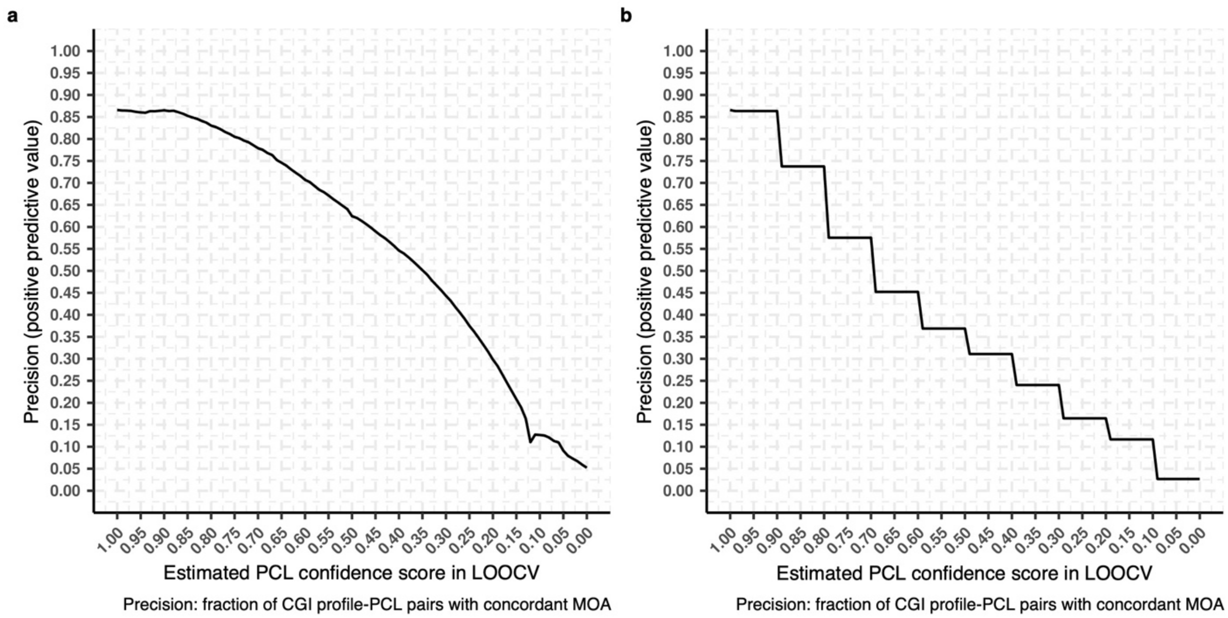

Supplementary Fig. 9 | Evaluating the predictive value of lower PCL confidence scores. In LOOCV, each reference set compound was withheld and CGI profiles from the remaining 436 reference set compounds were used in spectral clustering of each MOA to define PCLs, map PCL similarity score to PCL confidence score as in Supplementary Fig. 8, and finally estimate the PCL confidence scores for each CGI profile of the withheld reference set compound to every PCL cluster defined in its iteration of LOO. Across all (n = 437) iterations of LOOCV, the median number of PCLs was 1,117 and there were 10,470,531 CGI profile-PCL pairs with varying PCL confidence scores from which we could infer the predictive value of below high-confidence threshold (i.e., PCL confidence score < 1) similarities. Plotted are the proportion of reference set CGI profile-PCL pairs whose MOAs agreed that (**a**) had PCL confidence score ≥ X_i for each 0.01 increment or (**b**) had confidence score of 1, less than 1 to 0.9, less than 0.9 to 0.8, etc. for all bins. As expected, the accuracy of assigning a PCL's MOA to a particular CGI profile declined as the PCL confidence score declined. Matches with PCL confidence score ≥ 0.8 maintained relatively high MOA precision and appear acceptable for consideration when unknown compounds lack a high-confidence prediction and reference-based, MOA information is still of interest. LOOCV, leave-one-out cross-validation; CGI, chemical-genetic interaction; MOA, mechanism of action; PCL, Perturbagen Class. Source data are provided as a Source Data file.

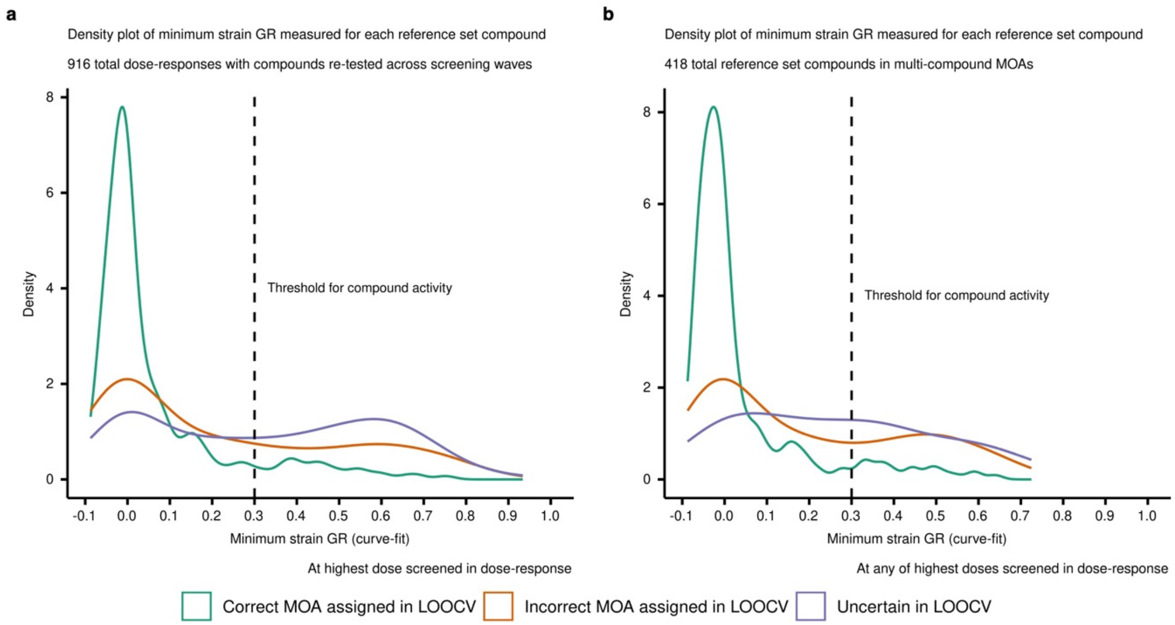

Supplementary Fig. 10 | Defining compound activity and distributions of minimum strain GR for reference set compounds. Reference set compounds that were assigned uncertain or incorrect MOA predictions in LOOCV tended to lack clear activity against any strain, whereas compounds that were correctly assigned often inhibited at least one strain completely. Defining a threshold for compound activity aided in identifying which reference and unknown compounds were most likely providing informative biological data, and which would be less trustworthy to make reference-based, MOA predictions for. **a**, Density plots of minimum strain GR achieved at each reference set compound's highest screening concentration in each of the screening waves (916 total dose-series from the 418 reference set compounds that were in multi-compound MOAs). Neglecting for demonstrable compound activity in the screen, the MOA of 501 screening-wave specific instances of reference set compounds would be correctly assigned in LOOCV (green), 187 would be incorrectly assigned (orange), and 228 would be uncertain (purple). **b**, Density plots of minimum strain GR achieved at each reference set compound's highest screening concentration over any one of the screening waves. Neglecting for demonstrable compound activity in any of the screens, 256 reference set compounds would be correctly assigned their MOA in LOOCV (green), while 118 would receive incorrect MOA assignments (orange) and 44 would be uncertain (purple). For both, the black dashed line (minimum strain GR = 0.3) is the threshold we defined for compound activity. Underlying GR scores shown were strain curve-fit GR values, as described in Supplementary Note 6, which reduced dose-to-dose noise; however, using raw GR values did not significantly change the overall observations. MOA, mechanism of action; LOOCV, leave-one-out cross-validation; GR, compound-induced growth rate. Source data are provided as a Source Data file.

a

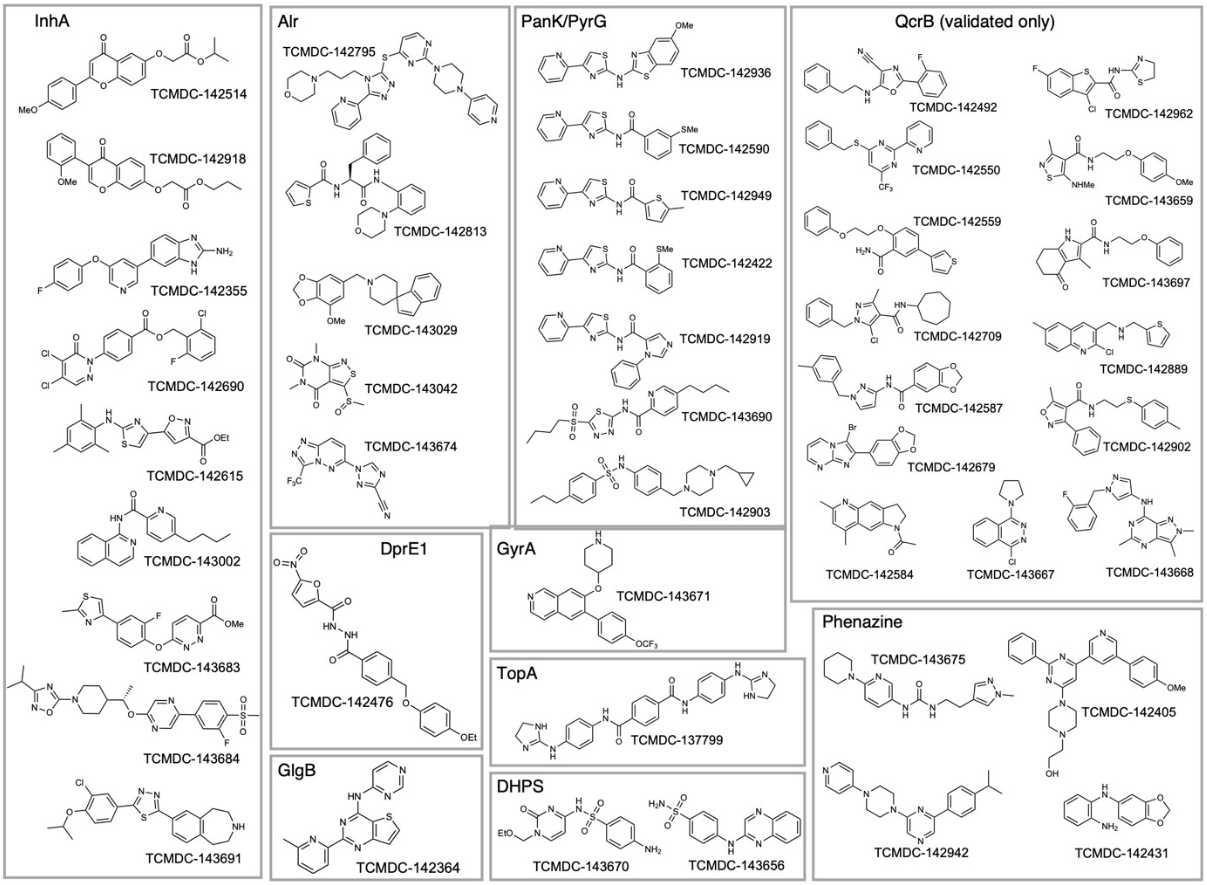

b

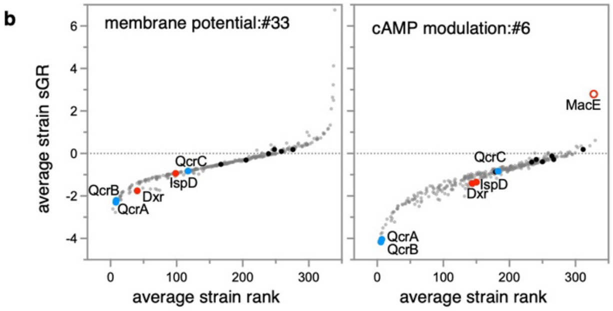

c

L2fc: sensitive −5 … 0 … 5 resistant; MIC shift

compound	class	*cydA::Tn*	QcrB A317T	WT MIC, μM
TCMDC-142359	IMP	-0.5	3.0	0.1
TCMDC-142376	IMP	-1.6	3.5	0.3
TCMDC-142398	IMP	-2.1	1.9	0.8
TCMDC-142444	IMP	-1.2	3.6	0.5
TCMDC-142511	IMP	-1.3	5.0	0.2
TCMDC-142533	IMP	-0.7	4.5	0.3
TCMDC-142546	IMP	-1.4	4.2	0.7
TCMDC-142557	IMP	-0.5	3.5	0.6
TCMDC-142595	IMP	-0.9	4.7	0.5
TCMDC-142697	IMP	-1.3	3.3	0.5
TCMDC-142785	IMP	-2.3	4.4	2.4
TCMDC-142828	IMP	-0.3	5.9	0.8
TCMDC-142992	IMP	-0.5	4.9	0.4
TCMDC-142490	PIP	-1.2	4.6	0.3
TCMDC-142814	PIP	-1.5	2.8	5.7
TCMDC-143650	PIP	-0.6	6.2	0.2
TCMDC-143652	PIP	-1.1	5.1	0.4
TCMDC-143653	PIP	-1.1	3.9	1.7
TCMDC-143657	PIP	-0.6	4.0	3.2
TCMDC-143698	PIP	-0.7	5.1	0.9
TCMDC-142353	QOA	-3.8	1.4	1.2
TCMDC-142388	QOA	-1.4	2.6	2.0
TCMDC-142461	QOA	-2.0	2.8	7.0
TCMDC-142552	QOA	-1.7	2.8	14.6
TCMDC-142715	QOA	-1.0	5.9	0.8
TCMDC-142430	QZS	-1.2	2.9	1.7
TCMDC-142498	QZS	-2.5	3.7	0.3
TCMDC-142537	QZS	-1.2	3.1	0.2
TCMDC-142368	TAP	-1.6	2.6	1.0
TCMDC-142408	TAP	-1.2	2.9	3.4
TCMDC-142724	TAP	-2.2	2.9	3.4
TCMDC-142980	PAB	-2.3	3.1	10.6

control compounds

Q203	IMP	-1.1	5.8	15 nM
CFZ	PHEN	-0.8	-0.3	0.6

Supplementary Fig. 11 | Structures of previously unannotated GSK TB set compounds with high-confidence MOA predictions and additional QcrB characterization. a, Structures of previously

unannotated GlaxoSmithKline (GSK) TB set compounds with high-confidence MOA predictions. Only high-confidence predictions (confidence score = 1) are shown. QcrB predictions not supported by MIC shifts in the *cydA*::Tn and *qcrB*$_{A317T}$ mutants are excluded. **b,** MOA-specific sensitization of cytochrome *bc1* components (QcrA and QcrB) in PCLs formed by non-QcrB-targeting respiration inhibitors. Standardized growth rate (sGR), for each strain, averaged over all compound-dose conditions in the PCL cluster is shown on the y-axis, whereas the average strain rank (based on sGR, 1= most sensitive, 340 = least sensitive) is shown on the x-axis. The PCL membrane potential:#33 is composed of 4 CGI profiles representing consecutive doses of CCCP, and cAMP modulation:#6 is composed of 5 CGI profiles representing consecutive doses of V-12-007960, which has been shown to inhibit adenylate cyclase activity. Cytochrome *bcc* components are highlighted in blue, isoprenoid synthetic enzymes in solid red, and solid black circles are wild-type controls. The MacE hypomorph (open red circle), depleted of an essential adenylate cyclase, shows relative resistance to V-12-007960. **c**, MIC shifts to annotated GSK QcrB inhibitors and controls: Q203 (QcrB inhibitor) and clofazimine in *cydA*::Tn and QcrB A317T mutants relative to wild-type (WT) H37Rv, similar to as shown in Fig. 3. All 32 of the previously annotated GSK QcrB inhibitors were correctly predicted to target QcrB via PROSPECT and PCL analysis (Supplementary Data 5). MOA, mechanism of action; PCL, Perturbagen Class; CGI, chemical-genetic interaction; L2fc, log2-fold change; MIC, minimum inhibitory concentration. Source data are provided as a Source Data file.

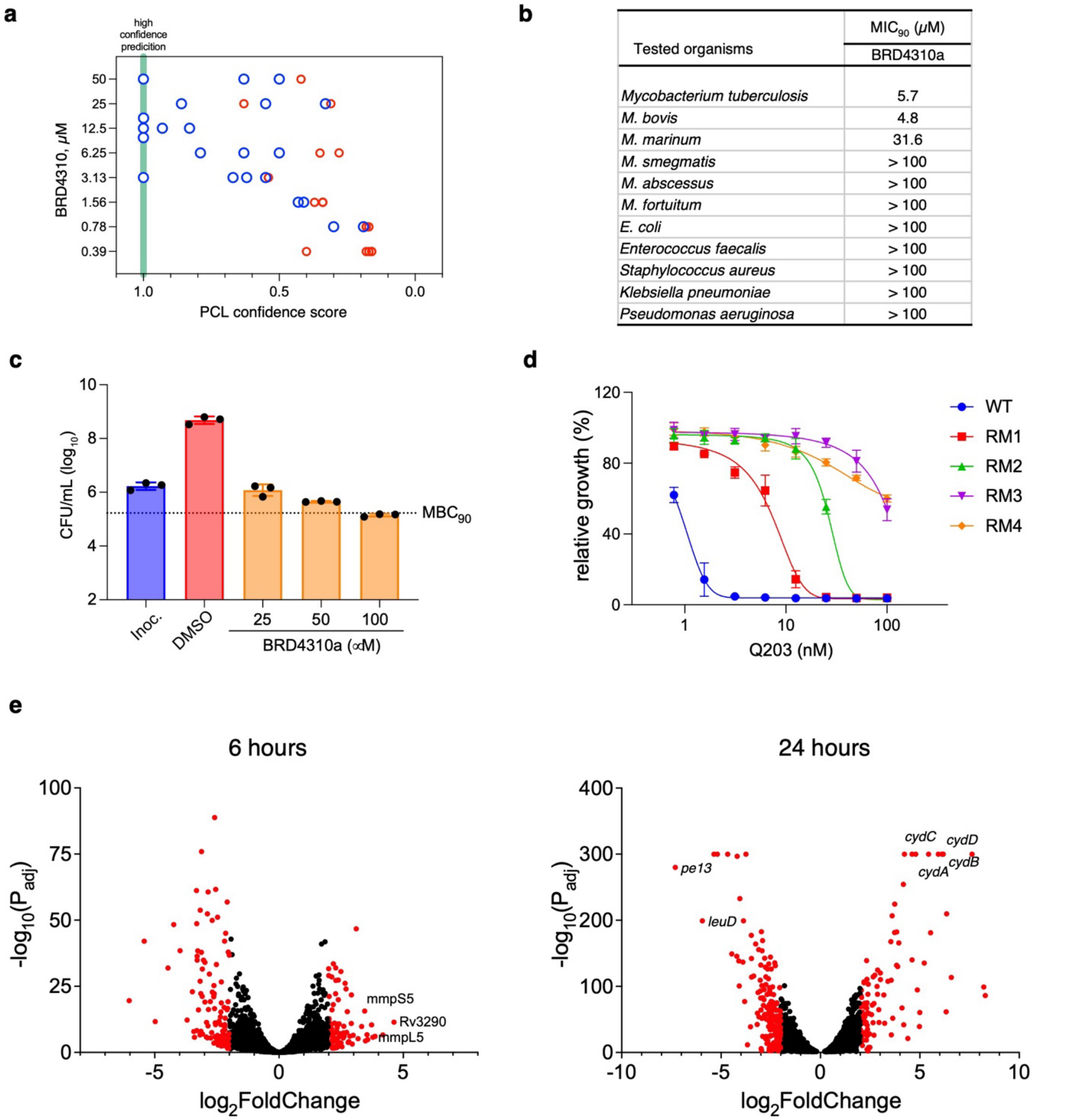

Tested organisms	MIC_{90} (μM) BRD4310a
Mycobacterium tuberculosis	5.7
M. bovis	4.8
M. marinum	31.6
M. smegmatis	> 100
M. abscessus	> 100
M. fortuitum	> 100
E. coli	> 100
Enterococcus faecalis	> 100
Staphylococcus aureus	> 100
Klebsiella pneumoniae	> 100
Pseudomonas aeruginosa	> 100

Supplementary Fig. 12 | QcrB MOA assignment and support for BRD4310 and BRD4310a. a, Dot plot of PCL confidence scores (X-axis, green line = 1, high confidence) for the top 5 ranking PCLs at each dose of BRD4310, with QcrB PCLs shown in blue and PCLs of other MOAs shown in red. The three high confidence PCL hits are highlighted: QcrB:#25, QcrB:#22, and QcrB:#11. **b**, MIC_{90} of BRD4310a against selected bacteria. **c**, Bactericidal activity of BRD4310a against wild-type H37Rv. Each bar plot shows the mean bacterial burden from biological triplicates (*n* = 3 independent experiments) and the error bars represent the standard deviation. The dotted line represents 90% bacterial killing compared with the initial inoculum (MBC_{90}). All experiments were performed in triplicate and repeated at least once. **d**, Dose–response curves of Q203 against wild-type (WT; blue) H37Rv and BRD4310a-resistant mutants (RMs): RM1 (red), RM2 (green), RM3 (purple), and RM4 (orange). Data are means of triplicates ± SD (*n* = 3 independent

experiments). BRD4310a-resistant mutant QcrB amino acid changes, nucleotide changes, and MIC fold change relative to H37Rv are shown in Fig. 4d. **e**, Volcano plot of differential gene expression of wild-type H37Rv exposed to BRD4310a at 6 h and 24 h. Each point represents the average value of one transcript across three replicates (*n* = 3). This was repeated *n* = 2 independent times with similar results. Differentially expressed genes (log2-fold change [log2FC] ≥ 2, adjusted P-value [Padj] of ≤ 0.05) are shown in red; all other genes appear in black. MOA, mechanism of action; PCL, Perturbagen Class; MIC, minimum inhibitory concentration; MBC, minimum bactericidal concentration. Source data are provided as a Source Data file.

Supplementary Table 1 | Accuracy statistics for MOA predictions on validatable, reference compounds in LOOCV.

Method	Reference compound activity filter	Total number of reference compounds evaluated	Number of compounds assigned correct MOA	Number of compounds assigned incorrect MOA	Number of uncertain compounds	Precision	Sensitivity	F1 score
PCL analysis[a]	Active and inactive	418	256	118	44	68%	61%	0.65
PCL analysis[a]	Active	337	235	77	25	75%	70%	0.72
PCL analysis[a]	Inactive	81	21	41	19	34%	26%	0.29
1-nearest neighbor[b]	Active	337	215	122	N/A	64%	64%	0.64
1-nearest neighbor[b]	Inactive	81	18	63	N/A	22%	22%	0.22
PCL analysis minus spectral clustering[c]	Active	337	37	17	283	69%	11%	0.19
PCL analysis minus individualized similarity score thresholds[d]	Active	337	123	34	180	78%	36%	0.50
Most frequent class[e]	Active	337	49	288	N/A	15%	15%	0.15
Random class assignment (average over 1,000 trials)[f]	Active	337	23	314	N/A	6.8%	6.8%	0.07

MOA, mechanism of action; LOOCV, leave-one-out cross-validation; CGI, chemical-genetic interaction. Only compounds in multi-compound MOAs can be assigned to the correct mechanism in LOOCV and are considered validatable. Compounds were defined as active if at least one strain had GR ≤ 0.3 at their highest screened concentration. Micro-averaged precision, sensitivity, and F1 score rounded to two significant figures. Source data are provided as a Source Data file.

[a] In our PCL analysis method, compounds were assigned an MOA if their similarity score surpassed a PCL's high-confidence similarity score threshold (i.e., PCL confidence score = 1), otherwise they were considered uncertain. In any case of ties between PCLs of different MOA, the tiebreaker rules selected the PCL for which the highest number of compound-doses (CGI profiles) of the query compound supported the assignment and, if needed, the highest PCL similarity score. For source data, see Supplementary Data 4.
[b] For the 1-nearest neighbor analysis, each compound was queried against the CGI profiles from all other compounds in the reference set and assigned the MOA of the most similar (highest pairwise Pearson correlation) CGI profile over any of its screened doses.
[c] When taking the complete set of CGI profiles from each MOA as one cluster per MOA instead of applying spectral clustering, 30 clusters/MOAs were predictive and defined as PCL clusters given that the reference set CGI profile with the highest similarity score to the set was in-MOA, while the other 41 were considered non-predictive as the reference set CGI profiles with the highest similarity score were out-of-MOA (see Fig. 2c). Compounds were assigned an MOA if their similarity score surpassed a PCL cluster's high-confidence similarity score threshold (i.e., PCL confidence score = 1), otherwise they were considered uncertain.
[d] Each compound's reference-based MOA prediction was selected as the PCL that it had the highest PCL similarity score to in LOOCV, rather than the highest PCL confidence score. Balancing precision and sensitivity equally, ROC analysis was performed, using the set of PCL similarity scores from each of the reference set compounds and binary labels (1 = agree, 0 = disagree) if the PCL matched the annotated MOA of the compound, to find the optimal similarity score threshold above which a known or unknown compound would be assigned the PCL's MOA with high-confidence or otherwise considered uncertain. Compounds were assigned an MOA if their similarity score surpassed the screen-wide similarity score threshold found in the ROC analysis (e.g., PCL similarity score ≥ 0.741 found for the set of active reference compounds), otherwise they were considered uncertain. Note that the similarity score threshold found in this

approach was more stringent than the high-confidence similarity score thresholds found and defined for most PCLs on an individualized basis (see Supplementary Fig. 7), contributing to significantly lower sensitivity in LOOCV compared to the full PCL analysis.

[e] Trivial model for baseline comparison where each compound's MOA prediction was selected as the most frequent MOA in the reference set: GyrAB.

[f] Trivial model for baseline comparison where each compound's MOA prediction was sampled randomly with replacement from their set (n = 337) of annotated MOAs, maintaining the same imbalanced MOA/class distributions. This was performed and accuracy statistics averaged over 1,000 trials. If the MOA distribution was balanced, the expected average accuracy would be 1 / N for multi-class classification over N classes or 1 / 48 = 2.1% in this case for the active reference compounds.

Supplementary Table 2 | Accuracy statistics for MOA predictions on annotated GSK set and new PCL predicted, experimentally validated QcrB inhibitors.

Method	Total number of compounds evaluated	Number of compounds assigned correct MOA	Number of compounds assigned incorrect MOA	Number of uncertain compounds	Precision	Sensitivity	F1 score
PCL analysis[a]	97	72	10	15	88%	74%	0.80
Tanimoto similarity[b]	95	36	59	N/A	38%	38%	0.38

MOA, mechanism of action; CGI, chemical-genetic interaction; GSK, GlaxoSmithKline. Micro-averaged precision, sensitivity, and F1 score rounded to two significant figures. Compounds include the 73 from the GSK set with previous MOA annotation, the 23 from the GSK set (21 unannotated and two annotated to target DHFR) that were predicted by PROSPECT and PCL analysis to target QcrB and tested against two QcrB MOA identifying mutant strains, and BRD4310. For the annotated GSK, compounds were correctly assigned if the MOA prediction from PCL analysis was concordant with the existing published MOA. For the 21 unannotated GSK predicted to share MOA with reference QcrB inhibitors, the 19 that showed shifts in MIC between WT, the QcrB resistant mutant strain, and *cydA*::Tn strain consistent with QcrB inhibition were tallied as correct MOA assignments. BRD4310 was tallied as a correct QcrB MOA assignment as predicted by PCL analysis, but not via Tanimoto similarity to the reference set. Source data are provided as a Source Data file.

[a] In our PCL analysis method, compounds were assigned an MOA if their similarity score surpassed a PCL's high-confidence similarity score threshold (i.e., PCL confidence score = 1), otherwise they were considered uncertain. In any case of ties between PCLs of different MOA, the tiebreaker rules selected the PCL for which the highest number of compound-doses (CGI profiles) of the query compound supported the assignment and, if needed, the highest PCL similarity score. For GSK set source data, see Supplementary Data 5 and, for BRD4310, Supplementary Fig. 12a.

[b] Trivial model for baseline comparison where each compound's inferred MOA was selected only based on its chemical structure as the reference set compound with the highest Tanimoto similarity to it. The two unannotated GSK compounds that were PCL predicted to target QcrB but failed strain validation are excluded as their exact MOA is still unknown. For source data, see Supplementary Data 6.

Supplementary Table 3 | Accuracy statistics for PCL-based MOA predictions on reference set in full model (fit to data/training).

	Total number of reference set compounds or conditions evaluated	Number of correct MOA assignments	Number of incorrect MOA assignments	Number of uncertain assignments	Precision	Sensitivity	F1 score
All reference set compounds (pert_id)	437	411	0	26	100%	94%	0.97
All batches of reference set compounds (broad_id)	505	477	0	28	100%	94%	0.97
All screening wave instances of batches of reference set compounds (proj_broad_id)	967	850	0	117	100%	88%	0.94
All reference set conditions/CGI profiles	9427	3840	0	5587	100%	41%	0.58

MOA, mechanism of action; pert_id, unique identifier for compound based on structure; broad_id, unique identifier for commercial or synthetic lot of a compound; proj_broad_id, unique identifier for an instance of a specific lot of compound screened in a particular screening wave; CGI, chemical-genetic interaction. Compounds or conditions were assigned an MOA if their similarity score surpassed the PCL's high-confidence similarity score threshold (i.e., PCL confidence score = 1), otherwise they were considered uncertain. The full model refers to the PCLs, high-confidence similarity score thresholds, and mappings of PCL similarity score to PCL confidence score for each PCL that were fit (or learned) using all reference set compounds and applied to all blinded test and unknown compounds. All PCL similarity scores and the true MOA labels for each of the reference set compounds was known when constructing the classifier and thus these statistics on reference set demonstrates the PCL analysis method's fit to and separation of MOAs in the data, not how well to expect the reference-based classification method to generalize to unseen compounds and data. Micro-averaged precision, sensitivity, and F1 score rounded to two significant figures. Source data are provided as a Source Data file.

Supplementary Table 4 | Accuracy statistics for MOA predictions on active, validatable, reference compounds in LOOCV broken down by existing, published strength of evidence for MOA annotation.

Strength of evidence	Total number of compounds evaluated	Number of compounds assigned correct MOA	Number of compounds assigned incorrect MOA	Number of uncertain compounds	Precision	Sensitivity	F1 score
Strong[a]	170	149	17	4	90%	88%	0.89
Moderate[b]	94	66	20	8	77%	70%	0.73
Weak[c]	73	20	40	13	33%	27%	0.30

MOA, mechanism of action; LOOCV, leave-one-out cross-validation; CGI, chemical-genetic interaction. Only compounds in multi-compound MOAs can be assigned to the correct mechanism in LOOCV and are considered validatable. Compounds were defined as active if at least one strain had GR ≤ 0.3 at their highest screened concentration. Micro-averaged precision, sensitivity, and F1 score rounded to two significant figures. Source data are provided as a Source Data file.

In our PCL analysis method, compounds were assigned an MOA if their similarity score surpassed a PCL's high-confidence similarity score threshold (i.e., PCL confidence score = 1), otherwise they were considered uncertain. In any case of ties between PCLs of different MOA, the tiebreaker rules selected the PCL for which the highest number of compound-doses (CGI profiles) of the query compound supported the assignment and, if needed, the highest PCL similarity score. For source data, see Supplementary Data 4.

[a] Strong; Reference compounds were annotated to have the strongest existing evidence for their MOA annotation in Mtb if published support included one or more lines of whole-cell genetic and biochemical evidence, including Mtb protein-compound co-crystal or cryo-EM structures, Mtb genetic mutations conferring resistance to compound treatment, Mtb-specific enzymatic assay inhibition, metabolite supplementation, etc or was a broad-spectrum antibiotic with well-validated whole-cell mechanistic evidence across one or more model bacterial organisms.
[b] Moderate; Reference compounds were annotated to have moderate existing evidence for their MOA annotation in Mtb if published support was limited to (1) whole-cell genetic and biochemical evidence for the protein target in other, select bacteria only while whole-cell growth inhibition was reported or likely (close analog of other known inhibitor) in H37Rv, (2) biochemical evidence that may be non-specific in the context of whole-cell activity (e.g., target-specific enzymatic assay and/or shifts in MIC in a Mtb strain underexpressing an essential protein), or (3) close analog of an inhibitor with strong, whole-cell MOA validation in Mtb but wasn't directly studied and reported itself.
[c] Weak; Reference compounds were annotated to have weak existing evidence for their MOA annotation in Mtb if published support was limited primarily to (1) non whole-cell, target-based enzymatic assay or computational docking or (2) whole-cell genetic and biochemical evidence for the protein target in other, select bacteria or eukaryotes only while they were found previously inactive in H37Rv.

Supplementary Note 1 | Primary analysis of barcode counts and log$_2$-fold change

After NGS of the PCR libraries, strain counts for each well were deconvoluted from the undemultiplexed main FASTQ files using the plate (P5 primer), well (P7 primer), and strain barcodes as previously described with the ConCensusMap script[1]. Spike-in control barcode counts in each well were used to log$_2$-normalize for coverage differences such that median PCR-control barcode counts were equal across wells with different P5 and P7 primers and between different PCR batches (explained in detail below). For every strain, compound, and compound dose combination, log$_2$(fold change) (L2FC) of spike-in control log-normalized strain counts compared to vehicle control wells were estimated using the ConCensusGLM package as previously described[5].

Each PCR plate had four unique P5 (plate) primers arrayed in quadrant formats (i.e., C2 for P5_1, C3 for P5_2, D2 for P5_3, D3_for P5_4 and so on) and sixty-six P7 (well) primers arrayed in square block format (i.e., C2-C3, D3-D4 for P7_1, …, M22-23, N22-23 for P7_66) which ensured every assay well had a unique P5-P7 primer combination for undemultiplexing strain counts. Using 25 distinct quartets of P5 primers, we were able to include up to 25 assay plates per pooled PCR library with 6,600 total unique P5-P7 primer pairs. The multi-lane architecture of the HiSeq 2500 flow cells enabled the sequencing of many PCR library pools at scale and repeated measurement of each P5-P7 primer pairs' relative efficiencies as estimated via counts of spike-in control barcodes.

Format of Arrayed P5 and P7 Primers for PCR Plates

	1	2	3	4	5	6	7	8	9	10	11	12	13	14	15	16	17	18	19	20	21	22	23	24
A																								
B																								
C		P5_1 P7_1	P5_2 P7_1	P5_1 P7_2	P5_2 P7_2	P5_1 P7_3	P5_2 P7_3	P5_1 P7_4	P5_2 P7_4	P5_1 P7_5	P5_2 P7_5	P5_1 P7_6	P5_2 P7_6	P5_1 P7_7	P5_2 P7_7	P5_1 P7_8	P5_2 P7_8	P5_1 P7_9	P5_2 P7_9	P5_1 P7_10	P5_2 P7_10	P5_1 P7_11	P5_2 P7_11	
D		P5_3 P7_1	P5_4 P7_1	P5_3 P7_2	P5_4 P7_2	P5_3 P7_3	P5_4 P7_3	P5_3 P7_4	P5_4 P7_4	P5_3 P7_5	P5_4 P7_5	P5_3 P7_6	P5_4 P7_6	P5_3 P7_7	P5_4 P7_7	P5_3 P7_8	P5_4 P7_8	P5_3 P7_9	P5_4 P7_9	P5_3 P7_10	P5_4 P7_10	P5_3 P7_11	P5_4 P7_11	
E		P5_1 P7_12	P5_2 P7_12	P5_1 P7_13	P5_2 P7_13	P5_1 P7_14	P5_2 P7_14	P5_1 P7_15	P5_2 P7_15	P5_1 P7_16	P5_2 P7_16	P5_1 P7_17	P5_2 P7_17	P5_1 P7_18	P5_2 P7_18	P5_1 P7_19	P5_2 P7_19	P5_1 P7_20	P5_2 P7_20	P5_1 P7_21	P5_2 P7_21	P5_1 P7_22	P5_2 P7_22	
F		P5_3 P7_12	P5_4 P7_12	P5_3 P7_13	P5_4 P7_13	P5_3 P7_14	P5_4 P7_14	P5_3 P7_15	P5_4 P7_15	P5_3 P7_16	P5_4 P7_16	P5_3 P7_17	P5_4 P7_17	P5_3 P7_18	P5_4 P7_18	P5_3 P7_19	P5_4 P7_19	P5_3 P7_20	P5_4 P7_20	P5_3 P7_21	P5_4 P7_21	P5_3 P7_22	P5_4 P7_22	
G		P5_1 P7_23	P5_2 P7_23	P5_1 P7_24	P5_2 P7_24	P5_1 P7_25	P5_2 P7_25	P5_1 P7_26	P5_2 P7_26	P5_1 P7_27	P5_2 P7_27	P5_1 P7_28	P5_2 P7_28	P5_1 P7_29	P5_2 P7_29	P5_1 P7_30	P5_2 P7_30	P5_1 P7_31	P5_2 P7_31	P5_1 P7_32	P5_2 P7_32	P5_1 P7_33	P5_2 P7_33	
H		P5_3 P7_23	P5_4 P7_23	P5_3 P7_24	P5_4 P7_24	P5_3 P7_25	P5_4 P7_25	P5_3 P7_26	P5_4 P7_26	P5_3 P7_27	P5_4 P7_27	P5_3 P7_28	P5_4 P7_28	P5_3 P7_29	P5_4 P7_29	P5_3 P7_30	P5_4 P7_30	P5_3 P7_31	P5_4 P7_31	P5_3 P7_32	P5_4 P7_32	P5_3 P7_33	P5_4 P7_33	
I		P5_1 P7_34	P5_2 P7_34	P5_1 P7_35	P5_2 P7_35	P5_1 P7_36	P5_2 P7_36	P5_1 P7_37	P5_2 P7_37	P5_1 P7_38	P5_2 P7_38	P5_1 P7_39	P5_2 P7_39	P5_1 P7_40	P5_2 P7_40	P5_1 P7_41	P5_2 P7_41	P5_1 P7_42	P5_2 P7_42	P5_1 P7_43	P5_2 P7_43	P5_1 P7_44	P5_2 P7_44	
J		P5_3 P7_34	P5_4 P7_34	P5_3 P7_35	P5_4 P7_35	P5_3 P7_36	P5_4 P7_36	P5_3 P7_37	P5_4 P7_37	P5_3 P7_38	P5_4 P7_38	P5_3 P7_39	P5_4 P7_39	P5_3 P7_40	P5_4 P7_40	P5_3 P7_41	P5_4 P7_41	P5_3 P7_42	P5_4 P7_42	P5_3 P7_43	P5_4 P7_43	P5_3 P7_44	P5_4 P7_44	
K		P5_1 P7_45	P5_2 P7_45	P5_1 P7_46	P5_2 P7_46	P5_1 P7_47	P5_2 P7_47	P5_1 P7_48	P5_2 P7_48	P5_1 P7_49	P5_2 P7_49	P5_1 P7_50	P5_2 P7_50	P5_1 P7_51	P5_2 P7_51	P5_1 P7_52	P5_2 P7_52	P5_1 P7_53	P5_2 P7_53	P5_1 P7_54	P5_2 P7_54	P5_1 P7_55	P5_2 P7_55	
L		P5_3 P7_45	P5_4 P7_45	P5_3 P7_46	P5_4 P7_46	P5_3 P7_47	P5_4 P7_47	P5_3 P7_48	P5_4 P7_48	P5_3 P7_49	P5_4 P7_49	P5_3 P7_50	P5_4 P7_50	P5_3 P7_51	P5_4 P7_51	P5_3 P7_52	P5_4 P7_52	P5_3 P7_53	P5_4 P7_53	P5_3 P7_54	P5_4 P7_54	P5_3 P7_55	P5_4 P7_55	
M		P5_1 P7_56	P5_2 P7_56	P5_1 P7_57	P5_2 P7_57	P5_1 P7_58	P5_2 P7_58	P5_1 P7_59	P5_2 P7_59	P5_1 P7_60	P5_2 P7_60	P5_1 P7_61	P5_2 P7_61	P5_1 P7_62	P5_2 P7_62	P5_1 P7_63	P5_2 P7_63	P5_1 P7_64	P5_2 P7_64	P5_1 P7_65	P5_2 P7_65	P5_1 P7_66	P5_2 P7_66	
N		P5_3 P7_56	P5_4 P7_56	P5_3 P7_57	P5_4 P7_57	P5_3 P7_58	P5_4 P7_58	P5_3 P7_59	P5_4 P7_59	P5_3 P7_60	P5_4 P7_60	P5_3 P7_61	P5_4 P7_61	P5_3 P7_62	P5_4 P7_62	P5_3 P7_63	P5_4 P7_63	P5_3 P7_64	P5_4 P7_64	P5_3 P7_65	P5_4 P7_65	P5_3 P7_66	P5_4 P7_66	
O																								
P																								

Well count coverage normalization was done in a three-step approach: (1) screening plates from each PCR batch were grouped, the log$_2$ differences between the median PCR-control count across the PCR batch and the median PCR-control count from each P5 primer (well quadrant) were found as the estimated coverage scaling factors so that all P5 primers would have equal coverage on average, and all strains counts log$_2$ shifted according to the coverage scaling factor for the P5

primer from that assay well then (2) wells were grouped by P5 primer, the $\log_2$ differences between the median PCR-control count for a P5 primer and the median PCR-control count for each P5-P7 primer pair were found as the next estimated coverage scaling factors so that all P5-P7 primers pairings for a P5 primer would have equal coverage on average, and all strain counts $\log_2$ shifted according to the coverage scaling factor for the P5-P7 primer pair from that assay well and finally (3) screening plates from each PCR batch were grouped, the $\log_2$ differences between the median PCR-control count across the PCR batch and the median PCR-control count from each P7 primer were found as the estimated coverage scaling factors so that all P7 primers would have equal coverage on average, and all strain counts $\log_2$ shifted according to the coverage scaling factor for the P7 primer from that assay well. This spike-in control $\log_2$-normalization technique corrected for systematic, reproducible reductions in coverage (total barcode counts) due to particular primer pairs which would appear downstream as strong compound activity and strain inhibition if unaccounted for.

Supplementary Note 2 | Growth rate score calculation from $\log_2$-fold change and strain quality control

Subsequent analyses and data visualization were performed in Matlab and R using the GCTx annotated data matrix format[2]. L2FC was calculated as previously[1]:

$$\mathrm{L2FC} = \log_2\left(\frac{\text{abundance}}{\text{abundance in vehicle}}\right)$$

Dose-dependent compound-induced growth rate (GR) inhibition was calculated from L2FC in reference to the onboard, positive control 256 nM or 498 nM rifampin treated wells which represented complete inhibition of the strain pool at the time of inoculation. Specifically, GR was calculated as:

$$\mathrm{GR} = 2^{\frac{\text{L2FC(rifampin)-L2FC(condition)}}{\text{L2FC(rifampin) - L2FC(vehicle)}}}\text{-}1$$

This is a transformation from the original equation for growth rate, $\mathrm{GR} = 2^{\log_2\left(\frac{x_{trt}(t)}{x(0)}\right)/\log_2\left(\frac{x_{DMSO}(t)}{x(0)}\right)}\text{-}1$ in Hafner et al.[3], with strain abundance in onboard, positive control rifampin taken as $x(0)$ to represent counts at time zero. A straightforward derivation from this definition of GR leads to its definition in the main text. While the resulting scale of GR for each strain was approximately between 0 (complete inhibition; rifampin) and 1 (uninhibited growth; vehicle), growth in a competitive, pooled format allowed strains to sometimes have a growth advantage in response to specific conditions and achieve GR > 1 (i.e., abundance and barcode counts greater than vehicle alone). Growth and measurements of GR > 1 is not strictly limited in this assay whereas growth inhibition and GR < 1 is limited more strictly around 0 due to the indestructability of DNA barcodes. GR measurements much greater than 1 were reproducible across biological replicates (i.e., strains had apparent growth advantages across replicate screening plates, multiple doses of the same compounds, multiple compounds of the same MOAs) and we believe reflected real biology (i.e., limited competition and fitness advantage in

the *Mtb* hypomorph pool). However, we anticipated that extreme, outlier values of GR >5 could be driven in part by a jackpotting effect during PCR and sequencing where the disproportionate, relative abundance of the strain in the pool was biologically meaningful, but the raw quantity of GR was not biologically reasonable and could interfere with downstream correlation-based analyses. To mitigate for the effect of very large strain GR values when later correlating conditions to one another, any strain GR >5 was re-scaled to GR = 5 + $\log_{10}$(GR).

For each screening wave independently, strains that grew slowly (i.e., L2FC(rifampin) > -1 indicating less than 1 baseline doubling) or unreliably (any conditions with GR > 20) were identified and filtered out from further analysis. Of the 387 strains that were included in the strain pool across all 6 screening waves, 42 strains were filtered out for slow growth in at least one of the screening waves and 5 strains were filtered out for high GR; the 340 strains that passed quality control were used in downstream analysis.

Strain category	Number of strains	Percent of strains
Used in PCL analysis	340	88%
Excluded – slow growth	42	11%
Excluded – noisy growth	5	1%
Excluded – not in all screening waves	84	-

Strains were excluded as slow growers if they achieved <1 doubling in one of the six screening waves (see Supplementary Data 2 for list). CarB, FolP1, MurD, PyrB, and H37RvBC02 were excluded as noisy growers due to many conditions with GR > 20.

Supplementary Note 3 | Standardized growth rate score calculation from GR

Each strain's condition GR distribution was first quantile normalized then robust z-scored using the publicly available CmapM Matlab package to calculate standardized growth rate (sGR), a metric of specific strain sensitivity or resistance relative to the median condition in the entire dataset[4,5]. For each condition and strain, where median(GR) and MAD(GR) are each approximately equal for all strains after quantile normalization:

$$\mathrm{sGR}_{\mathrm{treatment,strain}} = \frac{\mathrm{GR}_{\mathrm{treatment,strain}} - \mathrm{median}(\mathrm{GR}_{\mathrm{strain}})}{1.4826 \times \mathrm{MAD}(\mathrm{GR}_{\mathrm{strain}})}$$

GR quantile normalization, over all 340 strains, was performed using the quantilenorm Matlab function taking the median of the ranked values as described in Subramanian et al., in which the researchers similarly standardized expression profiles across cell lines prior to generating differential gene expression[5]. Standardizing the measured growth inhibition across strains according to this protocol served to mitigate mean-variance dependence in GR dependent on baseline strain growth rate which we observed would bias hit-calling and the rank order of strain inhibition to non-specific, completely inhibitory conditions towards how generally fit or quickly strains grew and not due to target-specific or systems biology-specific strain inhibition when

challenged with a particular dose of a chemical inhibitor (Supplementary Fig. 1d-f, Supplementary Fig. 3). The standardized metric of growth inhibition sGR was on similar scale and units for all strains such that the mean sGR across all conditions is 0 and standard deviation is 1, conditions with sGR much less than 0 indicated extreme strain inhibition relative to the bulk or average of all conditions screened, sGR much greater than 0 indicated extreme strain growth (or resistance) relative to the bulk of all screened conditions, and allowed for inference of each compound's or condition's biological mechanism from the rank order and magnitude of the sGR CGI profile with very little to any dependence on strains' differential growth rates. Quantile normalization and other rank-based and variance-stabilizing transformations have been similarly and commonly used across analysis of microarray and bulk and single-cell RNA-Seq datasets due to mean-variance-dependencies frequently arising from count data (in this case deep sequencing of strain DNA barcodes) that would otherwise make comparisons across cells lines (or strains) inaccurate[6-11]. Each condition's sGR profile (CGI profile) was the basic unit for further analysis making reference-based MOA predictions.

After the calculation of all robust z-score sGR values, CGI profile data for all reference set conditions were visualized using UMAP[12], implemented by the R package UMAP (v 0.2.7.0). Pearson distances between every pair of conditions were calculated as $\sqrt{(1 - \text{Pearson Correlation})}$. The UMAP embedding was performed with the seed set for the random_state and transform_state to 123, number of nearest neighbors for the initial embedding set to 20, and all other default parameters used.

Reference set compounds and doses considered fully inhibitory and included in Supplementary Fig. 3a-d:

Reference set compound	Pert ID	MOA	Number of doses considered fully inhibitory	Dose range considered fully inhibitory	Approximate *Mtb* MIC
Rifampin	BRD-K01507359	RpoB	15	0.0244 – 3.13	0.006
Rifapentine	BRD-A06889673	RpoB	6	1.56 – 50	0.39
Rifabutin	BRD-K43962705	RpoB	15	0.0244 – 50	0.006
Ciprofloxacin	BRD-K04804440	GyrAB	4	6.25 – 50	1.51
Levofloxacin	BRD-K09471561	GyrAB	5	3.13 – 50	0.69
Ofloxacin	BRD-A24228527	GyrAB	4	6.25 – 50	1.38
Isoniazid	BRD-K87202646	InhA	4	6.25 – 50	0.36
Q203	BRD-K59853741	QcrB	12	0.0061 – 12.5	0.01
BRD4592	BRD-K99844592	TrpAB	2	25 – 50	6.25

Concentrations shown are micromolar, most rounded to three significant figures. Expected *Mtb* MIC approximated based on published reports and in-house, absorbance-based MIC testing of H37Rv strain.

Supplementary Note 4 | Replicate correlation

For a descriptive metric of the data quality measured from each condition (screened in at least duplicate), replicate correlation (replicate reproducibility) was calculated as the average Spearman correlation between biological replicates of each condition on or across different assay plates. Specifically, for each well in a 384-well assay plate, strain counts were log2 transformed,

robust z-scored to their median counts in the 12 onboard vehicle control (DMSO) wells on each plate, correlated to replicates of that condition in other wells or plates, and the average Spearman rank correlation across replicates taken as the overall replicate reproducibility of the condition in the screen. Any conditions with only one replicate (due to technical error) were assigned null (NAN) replicate correlation values. Robust z-scoring and Spearman correlation calculation was performed using Matlab CmapM library functionality[5].

Supplementary Note 5 | Combining datasets across screening waves

All primary data analysis (strain barcode counts, L2FC, GR, sGR, and replicate correlation) was performed independently for each screening wave as their own screening datasets. These datasets were combined using GCTx format for further large-scale PCL analysis to include all conditions across the 6 screening waves and the 340 strains that passed quality-control in all waves[2]. For reference set compounds which were assayed in dose-response in at least two screening waves (one earlier against a ~400 strain pool and one later against a ~460 strain pool), the combined dataset had biological replicates (sGR CGI profiles from each individual screening wave) for each condition (compound-dose combination) which both aided in the reference-based PCL analysis and served as internal controls on the reproducibility of PROSPECT.

Supplementary Note 6 | GR curve-fitting and estimating compound activity

To estimate the activity level of each compound and dose of compound in PROSPECT, the drda R package [13] was used to find the best dose-response curve-fit for each strain's measured GR across a compound's dose series where GR of 1 represents no inhibition (vehicle) and GR of 0 represents full inhibition (256 nM or 498 nM rifampin). Quantile normalized GR was again used to mitigate the effects of strain-to-strain mean-variance dependency that could systematically bias the Hill slopes and upper and lower bounds of each strain's dose-response due to baseline growth rates. For each compound dose-series and strain, GR < 0 was truncated to 0, GR > 1 was compressed to be close to 1 (while maintaining rank order of the strains), and two dummy/null measurements of GR = 1 at 1/8 and 1/16 (3 and 4 dilutions below) the lowest assayed dose were concatenated to the observed GR measurements in order to aid in curve-fitting and more strictly constrain GR between 0 and 1.

$$\mathrm{GR}_{\text{compressed for curve−fitting}} = f(\mathrm{GR}) = \begin{cases} 0, & \mathrm{GR} < 0 \\ \mathrm{GR}, & 0 \le \mathrm{GR} \le 1 \\ 1 + \dfrac{\mathrm{GR} - 1}{10}, & \mathrm{GR} > 1 \end{cases}$$

The null datapoints (at concentrations very close to 0 uM, i.e., vehicle-like) incentivized the upper bound/asymptote on the dose-response model fits for each strain to be GR = 1 (uninhibited, vehicle-like growth) which is the simple and expected biological result had the compound been screened at a complete and infinitesimally dilute dose-series. Importantly, for strains that were significantly inhibited with GR < 1 across the entire dose-series of a compound, the use of the null datapoints aided in achieving monotonically decreasing functions that estimated return to

uninhibited growth with GR = 1 at some rate as compound concentration decreased unlike unconstrained best-fits, which could be monotonically increasing or nonmonotonic functions that are less biologically applicable and the result of overfitting to a limited dose-series. The null datapoints were included for dose-response curve fitting and otherwise excluded from all other analysis. 5-parameter logistic, 4-parameter logistic, and Gompertz function best-fit curves were found and the function with the lowest Akaike information criterion (AIC) was selected[14]. The values of the selected function at the compound's tested doses were taken as the strain's curve-fit, de-noised GR growth inhibition values and combined with all other strains to make up a curve-fit GR CGI profile for each assayed concentration of the compound.

Strain curve-fit GR, with the benefits of reducing dose-to-dose noise in the assay and stricter constraints on GR to be between 0 and 1, was then used to estimate compound activity as the lowest strain curve-fit GR achieved at the highest tested dose of the compound. Each condition's activity more generally was estimated as the lowest strain curve-fit GR achieved at each tested dose. Compounds that inhibited at least one strain significantly enough to achieve a curve-fit GR ≤ 0.3 at the highest tested dose (i.e., achieving a strain GR_{70} following Hafner et al.) were defined as active and all others that didn't meet this criterion were considered inactive (or minimally active) in the assay[3]. Using raw, quantile normalized GR and description of compound activity as the lowest GR achieved over any tested dose did not significantly change the proportion of compounds identified as active or inactive or the overall finding of higher MOA prediction sensitivity and precision for active compounds.

Supplementary Note 7 | Spectral clustering

To cluster sGR CGI profiles from each MOA category, we applied spectral clustering in Matlab, a robust technique that has been shown to segregate and cluster complex data more accurately than other common methods[15,16]. This process involved four steps. First, we calculated Pearson correlation as the similarity metric between condition sGR profiles. Second, we constructed a nearest-neighbor graph for each MOA class, connecting conditions to others within the same MOA that were within the top 20 closest conditions across all of the reference set (Supplementary Fig. 4). For each condition *i*, the correlation coefficients between it and all other conditions in the reference set were ranked such that rank 1 and rank 9,427 were respectively the highest correlated/most similar and lowest correlated/least similar conditions to condition *i* across all reference CGI profiles. For every pair of conditions *i* and *j*, their ranks in Pearson correlation were averaged to symmetrize the similarity matrix and if their average rank in Pearson correlation was ≤ 20 then the pair of conditions were connected as nearest neighbors. This is stored as an *n*-by-*n* adjacency matrix **W** where *n* is the number of conditions (CGI profiles) in the MOA and entries between connected conditions are 1 and disconnected conditions are 0. Third, we estimated the number of clusters *k* in each MOA by representing its similarity graph as a graph Laplacian and using an eigengap heuristic. The normalized symmetric Laplacian matrix (Ng-Jordan-Weiss) was calculated as $\mathbf{L_s}=\mathbf{D_g}^{-1/2}\mathbf{L}\mathbf{D_g}^{-1/2}$ where $\mathbf{L}=\mathbf{D_g}-\mathbf{W}$ and $\mathbf{D_g}$ is the degree matrix of **W**, an *n*-by-*n* diagonal matrix with entry $\mathbf{D_g}(i,i)$ equal to the total number of connected conditions in the MOA to condition *i* (i.e., the sum of row *i* in the adjacency matrix **W**)[15]. The eigenvectors and eigenvalues of the Laplacian matrix $\mathbf{L_s}$ were computed and the number of *k* clusters in the MOA were

estimated as the average (rounded up to the nearest integer) of $k_{zero\_plus\_one}$ and k_{gap} where $k_{zero\_plus\_one}$ is the index of the smallest non-zero eigenvalue and k_{gap} is the eigenvalue where the gap or change in consecutive eigenvalues was largest. While, in theory, the number of eigenvalues equal to zero k_{zero} indicates the number of connected components or clusters for perfectly separable data, real-world data is noisy and $k_{zero\_plus\_one}$ and k_{gap} have each been proposed as appropriate estimates for the number of connected components and termed spectral gap or eigengap heuristics[15,16]. We found that estimating k as the average of $k_{zero\_plus\_one}$ and k_{gap} resulted in the greatest overall performance in our method. We reasoned that this could be due to $k_{zero\_plus\_one}$ underfitting for some MOAs resulting in too few and large of clusters and k_{gap} overfitting for some MOAs resulting in too many and small of clusters. Ultimately, this allowed for estimation of the number of k clusters in each MOA to be fully automated and standardized. Finally, using the spectralcluster Matlab function, for each MOA and its estimated k, the k clusters were found by reducing the condition space (n-by-n) to a lower-dimensional (n-by-k) space using the first k eigenvectors of the Laplacian matrix and assigning each condition i in n to one of k clusters using k-means clustering[17,18]. Specifically, the first k eigenvectors of $\mathbf{L_s}$ are combined column-wise into an n-by-k matrix $\mathbf{V}$ and each row of $\mathbf{V}$ is normalized to have unit length. The n points (conditions) are then clustered in the embedded k-dimensional feature space using k-means clustering and assigned into one of k clusters. This process was repeated for each MOA separately and the cluster identifiers and CGI profiles members of each cluster were stored in GMT file format. For reproducibility, the default Matlab random number generator (i.e., Mersenne Twister generator with seed 0) was initialized using the rng function, the maximum number of computational threads was set to 4 using the maxNumCompThreads function, and the order of conditions in the $\mathbf{W}$, $\mathbf{D_g}$, $\mathbf{L}$, and $\mathbf{L_s}$ matrices was sorted by hierarchical clustering of the Pearson correlation matrix using CmapM's hclust function for each MOA's iteration of spectral clustering.

Supplementary Note 8 | PCL cluster definition and similarity score

We defined and calculated a PCL similarity score between each test CGI profile and each cluster from unsupervised, spectral clustering as the median of the Pearson correlation coefficients between the test CGI profile and all reference set CGI profiles within the cluster. Clusters whose highest similarity scoring, reference set CGI profile shared the same annotated MOA as the cluster members (i.e., one of the cluster CGI profiles themselves or a CGI profile from a compound in the same MOA but was a member of a different cluster) were kept for use in making MOA predictions and defined as PCLs. However, clusters whose highest similarity scoring reference set CGI profile was from a compound of a different annotated MOA than the cluster members were discarded as non-predictive clusters whose pattern of strain inhibition was non-specific to one single MOA and therefore were difficult to interpret for predicting MOA on unknown compounds. When calculating PCL similarity score between a reference set CGI profile and a cluster it was a member of, self-similarity (Pearson correlation of 1) was excluded from the median calculation. Including self-similarity in this calculation would unfairly bias higher similarity scores for CGI profiles in the cluster than CGI profiles from new, unknown compounds whose cluster membership is unknown (had their MOAs been annotated and included in the reference set) and reference set CGI profiles that belong to other clusters and would otherwise bias comparison of similarity scores when

defining the thresholds to be used for making high-confidence MOA predictions. PCL similarity scores for each CGI profile to each PCL were stored in GCTx annotated data matrix format.

Supplementary Note 9 | One-vs-rest (OvR) binary classification strategy

We posed MOA prediction on unknown compounds as a multi-class classification problem using a one-vs-rest binary classification strategy in which a single classifier per PCL is trained with reference set compounds of its MOA as positive samples and reference set compounds of all other MOAs as negative samples; when applied to an unknown compound it outputs a real-valued confidence score of the unknown compound acting via the same MOA as the PCL[19-21]. For each PCL, we had the set **S** = {(x_1, y_1), …, (x_{437}, y_{437})} of 437 reference set compound training samples where $x_i \in \mathbf{X}$ was the maximum similarity score of the *i*th compound (over all of its CGI profiles, i.e, doses) to the PCL, $y_i \in \mathbf{Y}$ is 1 if the compound shares the same MOA as the PCL and 0 otherwise. Each PCL's classifier is a function that maps a similarity score x to a confidence score (ranging from 0 to 1) estimating the likelihood of the compound acting via the PCL's annotated MOA. Specifically, it is the proportion of reference set compounds with similarity score ≥ x that share the PCL's MOA, i.e., the precision (or positive predictive value, i.e. PPV) of MOA assignment if the similarity score x was used as the threshold above which all reference set compounds are labeled 1 and all reference set compounds below are labeled 0. Confidence scores (i.e., PPV) at each x_1, ..., x_{437} were computed using perfcurve in Matlab. While the initial mapping was calculated using the highest similarity scores from each of the 437 compounds to avoid repeated tallying of non-independent samples (multiple CGI profiles of the same compound), the mapping was extended to similarity scores from all reference set CGI profiles via next neighbor interpolation (interp1 in Matlab) – the confidence score for a similarity score from an unobserved CGI profile is the confidence score of the next highest similarity score from an observed CGI profile (i.e., the most similar profile for a reference set compound). The rationale follows: for an unobserved CGI profile from reference set compound *i* with similarity score $\bar{x}_i < x_i$ and lowest observed similarity score $x > \bar{x}_i$, replacement of ($\bar{x}_i$, y_i) for (x_i, y_i) in set **S** (e.g., the dose with similarity score x_i wasn't screened and $\bar{x}_i$ was instead the maximum similarity score of compound *i* to the PCL) during initial mapping would compute a confidence score for $\bar{x}_i$ equal to the confidence score for x. In other words, the proportion of reference set compounds with similarity scores ≥ $\bar{x}_i$ excluding x_i is the same as the proportion with similarity scores ≥ x including x_i. We reasoned that compounds would be the most appropriate, independent data points to tally as evidence supporting an MOA call for a PCL, as opposed to doses which could be from a single compound not shared across the MOA and lead to strong biases toward large MOAs (where the relative differences in the number of CGI profiles per MOA is ≈10 times greater than the number of compounds). The full mapping of similarity score to confidence score for every reference set CGI profile to every PCL was stored and referred to as the full model. High-confidence similarity score thresholds were defined for each PCL as the lowest similarity score where confidence score = 1.

Supplementary Note 10 | Applying PCL classifiers to CGI profiles from unknown compounds

When applying a PCL classifier to an unknown compound, the similarity scores of its CGI profiles were mapped to confidence scores via linear interpolation (interp1 in Matlab) using the mapping of similarity score to confidence score trained on the reference set. This approach considers the distance (or difference) to an unknown CGI profile's similarity score x and the two reference set CGI profile similarity scores it is between $x_n > x > x_m$ when estimating the confidence score for x based on the confidence scores of x_n and x_m. Confidence scores were estimated for every unknown CGI profile to every PCL and stored in GCTx annotated data matrix format.

Supplementary Note 11 | PCL-based MOA predictions for unknown compounds

For each unknown compound, its most likely MOA in the reference set was predicted as the PCL with the highest confidence score over one or more of its CGI profiles, following the typical one-versus-rest multi-class classification strategy[19-21]. Since there can be PCL classifiers (of the same or different MOAs) with equally high confidence scores for an unknown compound, we made one MOA prediction for every compound via a majority rules tiebreaker in which the PCL that had the highest confidence score over the greatest number of doses (CGI profiles) and highest similarity score overall, if necessary, was selected. The predicted MOA was considered a high-confidence MOA assignment if the PCL confidence score was equal to 1. A compound's MOA was considered uncertain if its highest PCL confidence score was below 1. For unknown compounds lacking a high-confidence MOA assignment, their predicted MOAs and the strength of their PCL confidence scores could still be considered, to provide mechanistic insight for more unknown compounds with slightly reduced confidence (Supplementary Fig. 9).

The above approach can be taken to separately predict MOA at every data level for a compound – dose (i.e., CGI profile), specific lot or batch of a compound in a particular screening wave (i.e., proj_broad_id identifier), specific lot or batch of a compound over all screening waves (i.e., broad_id identifier), or compound (i.e., pert_id identifier) – for comparing behavior between screens or commercial and synthetic batches of a compound (Supplementary Table 3, Supplementary Data 4). As well, for compounds with equally high confidence scores to PCLs from multiple MOAs, each MOA and the reference compounds from each of the PCLs can be considered holistically (i.e., multi-label classification) and motivate follow-up experimental validation and rule-out of each of the MOAs (Supplementary Data 5).

Supplementary Note 12 | Leave-one-out cross validation and tested PCL analysis variations

For each reference set compound, its CGI profiles were removed from the reference set and all CGI profiles from the remaining 436 reference set compounds were used for unsupervised clustering, PCL cluster definition and similarity scoring, and for training PCL classifiers to map similarity score to confidence score as described above. Using the mappings of similarity score to confidence score for each PCL, confidence scores for each of the left-out reference set compound's CGI profiles were estimated via linear interpolation, as described above for unknown compounds using the full model. The estimated confidence scores of each reference set compound to each PCL from its iteration of LOOCV were combined. A single MOA prediction was

made for each reference set compound based on the highest confidence scoring PCL (using the same majority rules tiebreaker as above) and considered to be a high-confidence MOA assignment if the confidence score was 1. True positives and false positives in LOOCV were tallied as the number of reference set compounds whose high-confidence MOA assignments were concordant or discordant, respectively, with their annotated MOAs and compounds that were uncertain (i.e., all PCL confidence scores < 1) were tallied as false negatives since their annotated MOAs were unable to be predicted with high-confidence. Micro-averaged precision, sensitivity, and F1 score statistics were calculated using the counts of true positives, false positives, and false negatives in LOOCV[22] with specific focus on reference set compounds that were identified as active in the screen (Supplementary Table 1). 19 compounds were the sole representative of their annotated MOAs in the reference set and cannot be correctly predicted in LOOCV – since no PCLs of their MOA or other data annotated to their MOA exist in the training set when they are withheld.

To evaluate the importance of the unsupervised clustering step in identifying highly similar clusters of CGI profiles within each MOA and filtering out less informative CGI profiles, we repeated the standard LOOCV procedure described above but, in each iteration, took the complete set of CGI profiles from each MOA as one comparison group per MOA instead of applying spectral clustering. The following steps of PCL cluster definition and similarity scoring, PCL classifier training, estimation of PCL confidence scores and MOA prediction for each left-out reference set compound, and calculation of micro-average accuracy statistics remained the same with up to 71 groups (one for each MOA) as the starting point instead of the clusters from spectral clustering (1,117 clusters on average) (Supplementary Table 1).

To evaluate the importance of the PCL classifier training step and defining high-confidence similarity score thresholds unique to each PCL using confidence score, we tested the standard LOOCV procedure described above but each left-out reference set compound's MOA prediction was made to the PCL with the highest similarity score over any of its CGI profiles. Balancing precision and sensitivity equally, ROC analysis was performed in R, using the set of PCL similarity scores from each of the reference set compounds and binary labels (1 = agree, 0 = disagree) if the PCL matched the annotated MOA of the compound, to find the optimal similarity score threshold above which a known or unknown compound would be assigned the PCL's MOA with high-confidence or otherwise considered uncertain[23]. The similarity score thresholds found in this approach were more stringent than the high-confidence similarity score thresholds found and defined for most PCLs on an individualized basis (Supplementary Table 1, Supplementary Fig. 7, 8). Compounds were assigned an MOA if their similarity score surpassed the screen-wide similarity score threshold found in the ROC analysis (e.g., PCL similarity score ≥ 0.649 found for reference set compounds from multi-compound MOAs), otherwise they were considered uncertain. The preceding steps of unsupervised clustering and PCL cluster definition and similarity scoring and the following step to calculate micro-average accuracy statistics were the same as the standard LOOCV procedure.

To further evaluate the benefit of the PCL analysis method and the corroboration of multiple similar reference set CGI profiles (PCL clusters) when comparing to test compounds, we evaluated

the predictive performance of a simple 1-nearest neighbor approach where the MOA assigned to a test compound is the MOA of the reference set CGI profile with the highest Pearson correlation coefficient in LOOCV. For each left-out reference set compound, all its CGI profiles were removed from the reference set and its 1-nearest-neighbor (over any of the left-out compound's screened compound-doses) was selected as the CGI profile from the remaining reference set with the highest Pearson correlation coefficient. The MOA of each compound's 1-nearest neighbor in LOOCV was compared to their annotated MOA to calculate the accuracy of the predictions. Micro-averaged precision, sensitivity, and F1 score statistics were calculated using the counts of true positives and false positives in LOOCV with specific focus on reference set compounds that were identified as active in the screen (Supplementary Table 1).

Supplementary Note 13 | Tanimoto similarity calculation

The pairwise Tanimoto similarity coefficient was calculated between each compound in the GSK set and BRD4310 and each compound in the reference set using the RDKit library (v.2020.09.1) in Python (v.3.7.12). For each compound, its SMILES was converted to an RDKit molecular object and its Morgan fingerprint was computed using a neighborhood size of 2 and all other default parameters[24]. The Tanimoto similarity coefficient was calculated for every pair of test and reference set molecular fingerprints. For each GSK compound and BRD4310, the reference compound with the highest Tanimoto similarity coefficient was taken as its nearest chemical neighbor in the reference set (Supplementary Table 2, Supplementary Data 6).

Supplementary Note 14 | Chemical synthesis and characterization

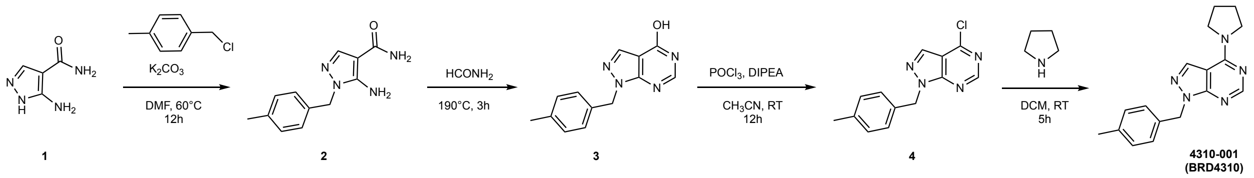

Synthesis of 4-(1-pyrrolidinyl)-1-[(p-tolyl)methyl]-1,2,5,7-tetraaza-1H-indene (**BRD4310**, i.e., **4310-001**):

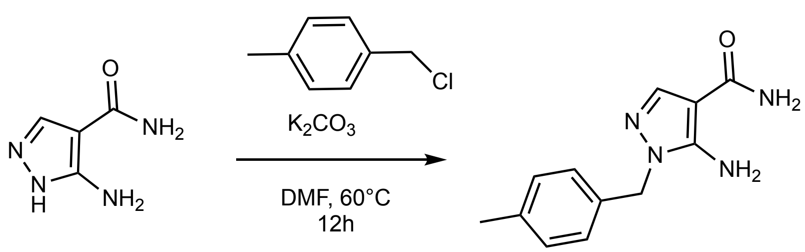

1 **2**

To a mixture of 5-amino-1H-pyrazole-4-carboxamide (5.00 g, 39.68 mmol, 1 eq) and K_2CO_3 (10.95 g, 79.37 mmol, 2 eq) in DMF (dimethylformamide, 198 mL) was added 1-(chloromethyl)-4-

methylbenzene (11.19 g, 39.68 mmol, 2 eq). The resulting mixture was stirred at 60 °C for 12 h. After being cooled to room temperature, the reaction mixture was diluted with water (50 mL) and extracted with EA (ethyl acetate, 50 mL×3). The combined organic phase was dried over anhydrous Na_2SO_4 and concentrated in vacuo. The residue was purified by silica gel chromatography to give 2.80 g of 5-amino-1-(4-methylbenzyl)-1H-pyrazole-4-carboxamide. Yield was 30%. LC-MS m/z calc. for $C_{12}H_{14}N_4O$ $[M+H]^+$ = 231.1, found: 231.4, t_R = 1.68 min.

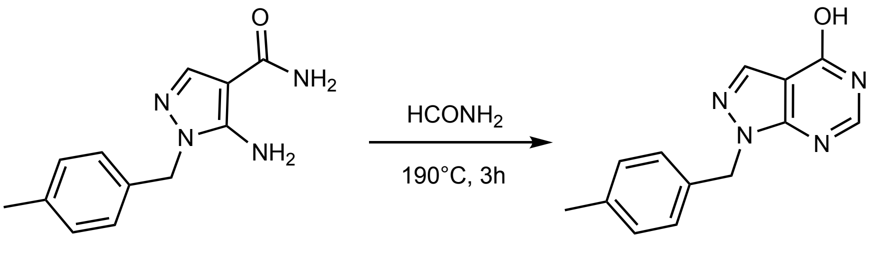

2 3

A mixture of 5-amino-1-(4-methylbenzyl)-1H-pyrazole-4-carboxamide (3.00 g, 13.02 mmol) and formamide (26 mL) was stirred at 190 °C for 3 h. After being cooled to room temperature, the reaction mixture was diluted with DCM (dichloromethane, 50 mL), washed with water (50 mL×3), dried over anhydrous Na_2SO_4 and concentrated in vacuo. The residue was purified by silica gel chromatography to give 2.30 g of 1-(4-methylbenzyl)-1H-pyrazolo[3,4-d]pyrimidin-4-ol. Yield was 73%. LC-MS m/z calc. for $C_{13}H_{12}N_4O$ $[M+H]^+$ = 241.1, found 241.1, t_R = 1.71 min.

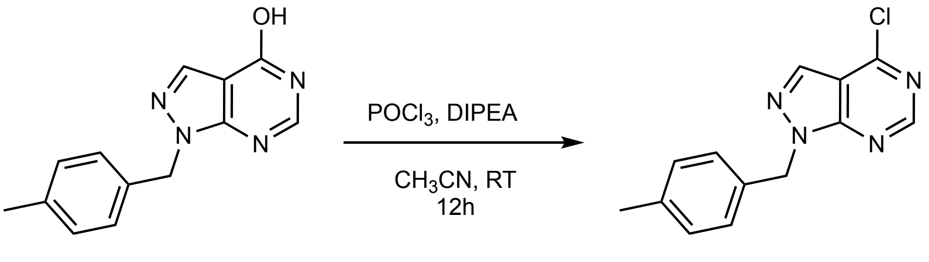

3 4

To a mixture of 1-(4-methylbenzyl)-1H-pyrazolo[3,4-d]pyrimidin-4-ol (1.00 g, 4.16 mmol, 1 eq) in CH_3CN (14 mL) were added $POCl_3$ (5.74 g, 37.42 mmol, 9 eq) and DIPEA (N,N-Diisopropylethylamine, 1.18 g, 9.15 mmol, 2.2 eq). The resulting mixture was stirred at 0 °C for 15 min and at room temperature for 12 h. The reaction mixture was diluted with CH_3CN (10 mL) and quenched with H_2O (10 mL). The resulting mixture was adjusted with $NaHCO_3$ to pH = 8. The mixture was extracted with DCM (dichloromethane, 100 mL). The extracts were washed with brine, dried over anhydrous Na_2SO_4 and concentrated in vacuo. The residue was purified by silica gel chromatography to give 900 mg of 4-chloro-1-(4-methylbenzyl)-1H-pyrazolo[3,4-d]pyrimidine. Yield was 83%. LC-MS m/z calc. for $C_{13}H_{11}ClN_4$ $[M+H]^+$ = 259.1, found 259.1, t_R = 1.71 min.

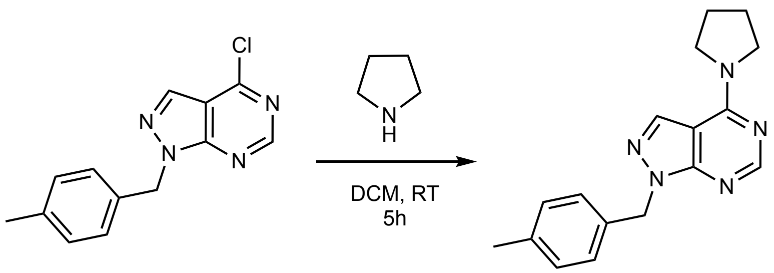

4 **4310-001**

To a mixture of 4-chloro-1-(4-methylbenzyl)-1H-pyrazolo[3,4-d]pyrimidine (300 mg, 1.16 mmol, 1 eq) in DCM (dichloromethane, 4 mL) was added pyrrolidine (1.94 mL, 23.25 mmol, 20 eq). The resulting mixture was stirred at room temperature for 5 h. The reaction mixture was diluted with water (20 mL) and extracted with EA (ethyl acetate, 30 mL×3). The combined organic phase was dried over anhydrous Na_2SO_4 and concentrated in vacuo. The residue was purified via prep-HPLC to provide **BRD4310** (i.e., **4310-001**) in 20% yield (68 mg). LC-MS m/z calc. for $C_{17}H_{19}N_5$ $[M+H]^+$ = 294.2, found 295.4, t_R = 1.86 min. ^1H NMR (400 MHz, DMSO-d_6) δ 8.37 (d, J = 9.9 Hz, 1H), 8.31 (s, 1H), 7.11 (d, J = 8.7 Hz, 4H), 5.50 (s, 2H), 3.84 (t, J = 6.7 Hz, 2H), 3.68 (t, J = 6.7 Hz, 2H), 2.25 (s, 3H), 2.09 (dt, J = 13.0, 6.5 Hz, 2H), 1.99 (dd, J = 13.1, 6.5 Hz, 2H).

BRD4310 ^{1}H NMR and LC-MS:

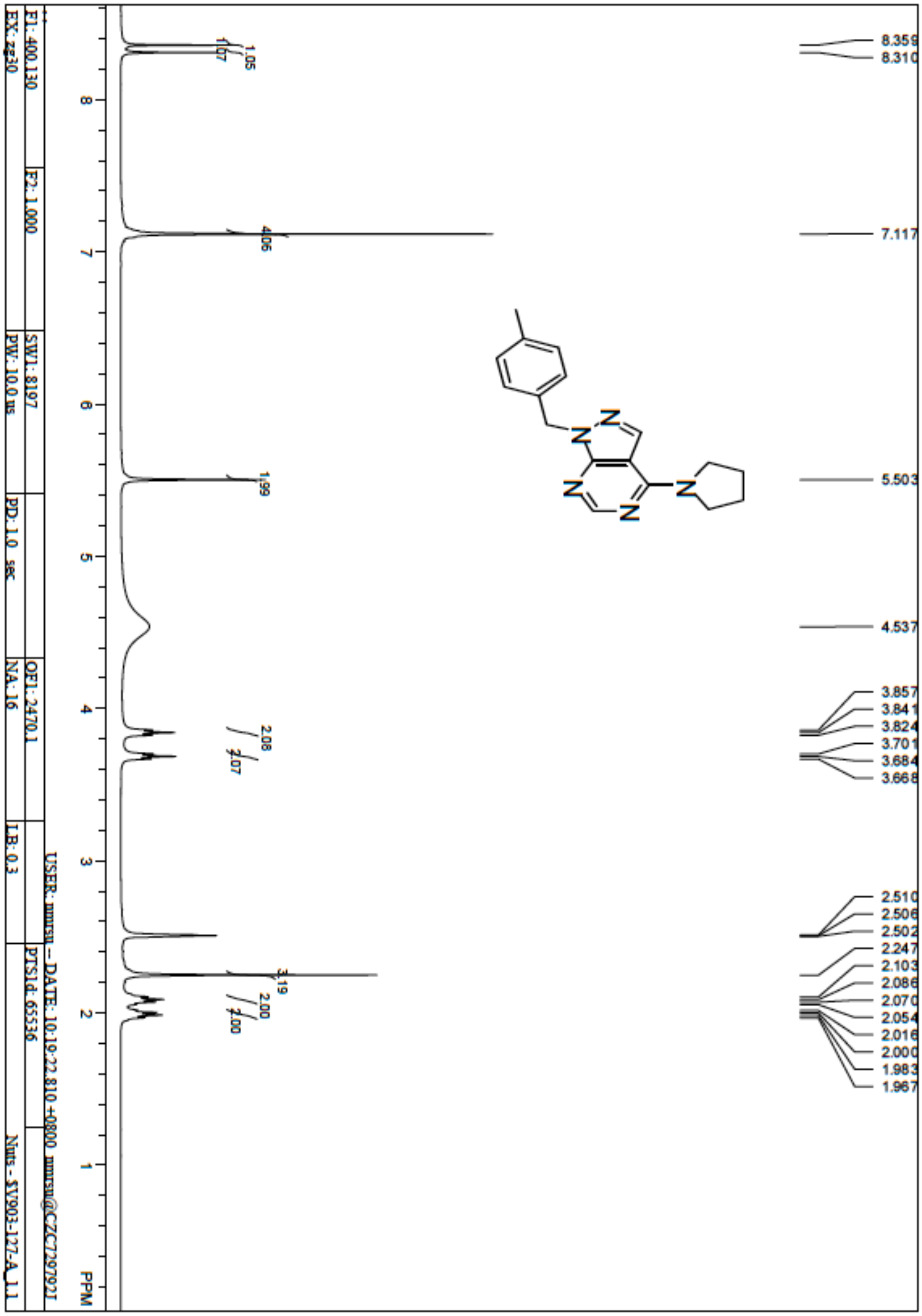

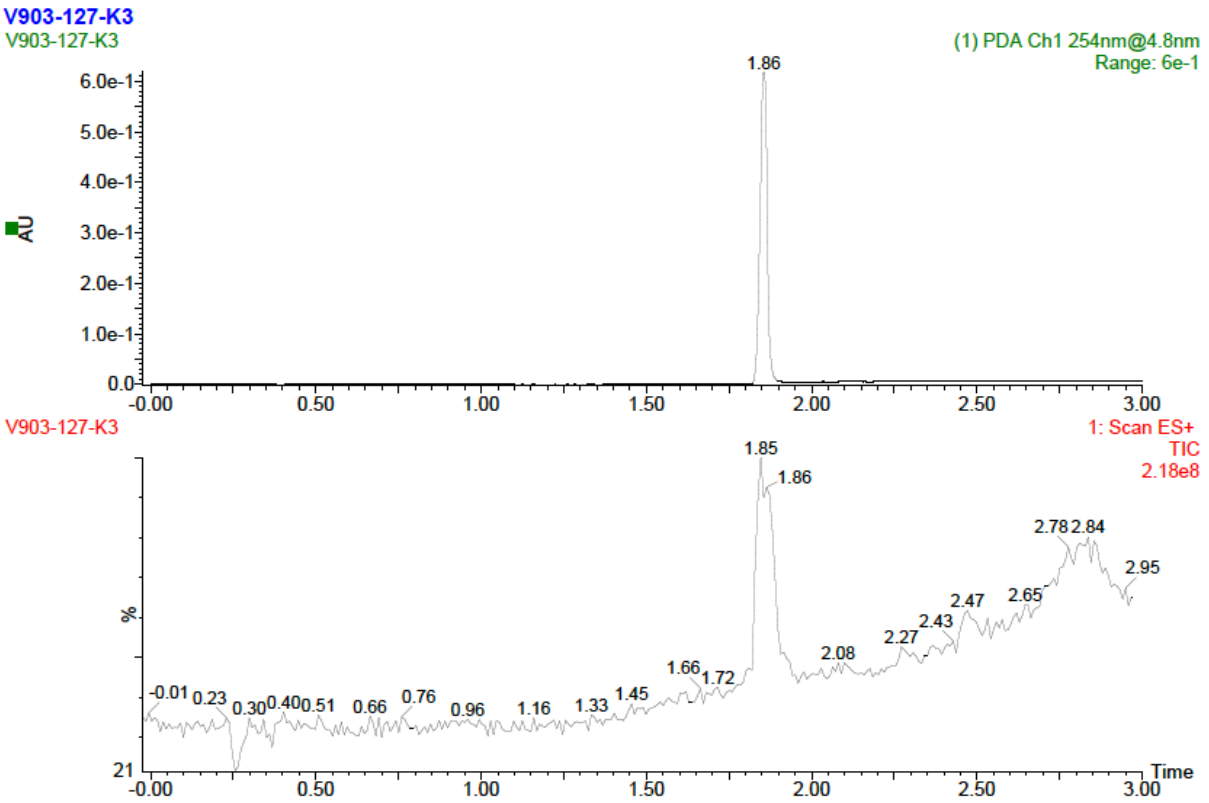
V903-127-K3
V903-127-K3
(1) PDA Ch1 254nm@4.8nm
Range: 6e-1
AU
1.86
V903-127-K3
1: Scan ES+
TIC
2.18e8
%
1.85
1.86
2.78 2.84
2.95
2.47
2.65
2.43
2.27
2.08
1.66
1.72
-0.01 0.23 0.30 0.40 0.51 0.66 0.76 0.96 1.16 1.33 1.45
Time

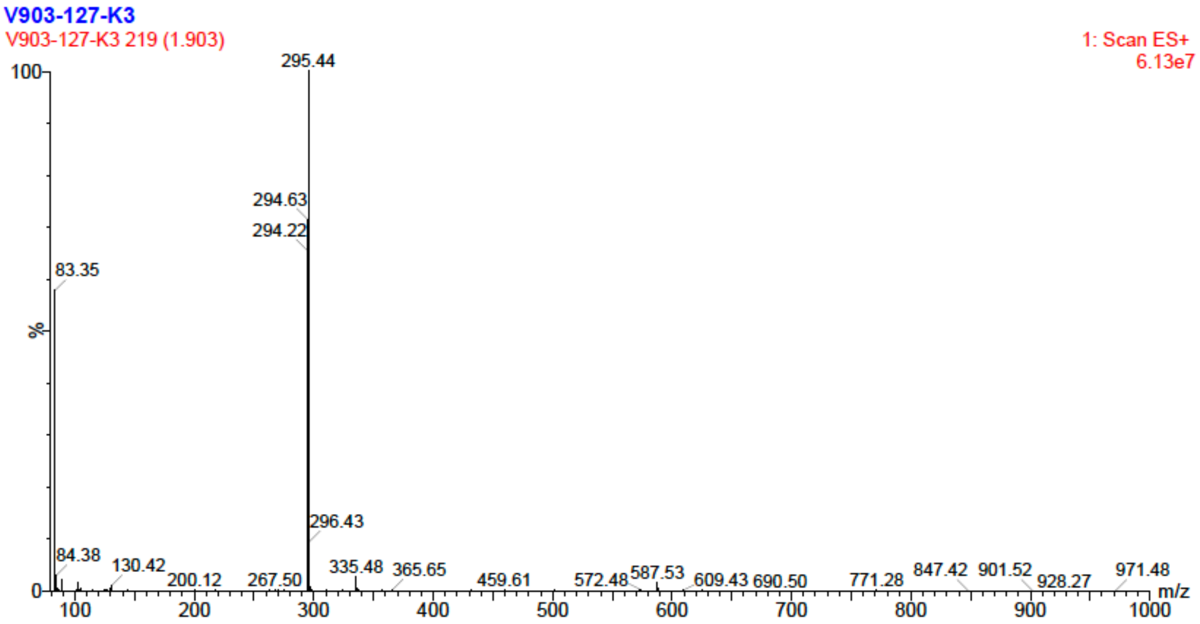
V903-127-K3
V903-127-K3 219 (1.903)
1: Scan ES+
6.13e7
%
295.44
294.63
294.22
83.35
296.43
84.38
130.42
200.12
267.50
335.48
365.65
459.61
572.48
587.53
609.43
690.50
771.28
847.42
901.52
928.27
971.48
m/z

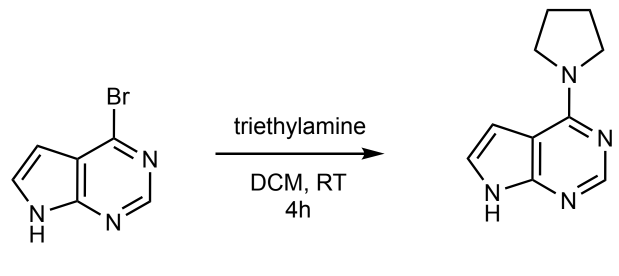

Intermediate B

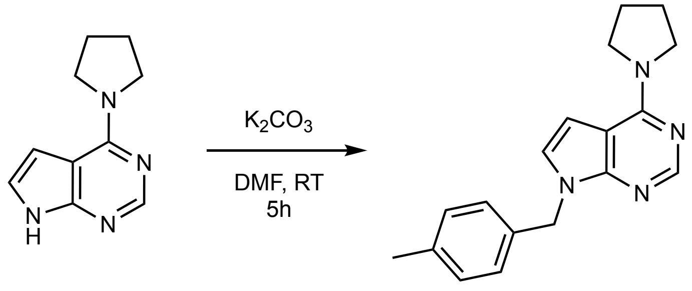

Intermediate B

4310-008
(BRD4310a)

Synthesis of 4-(1-pyrrolidinyl)-1-[(p-tolyl)methyl]-1,5,7-triaza-1H-indene (**BRD4310a**, i.e., **4310-008**):

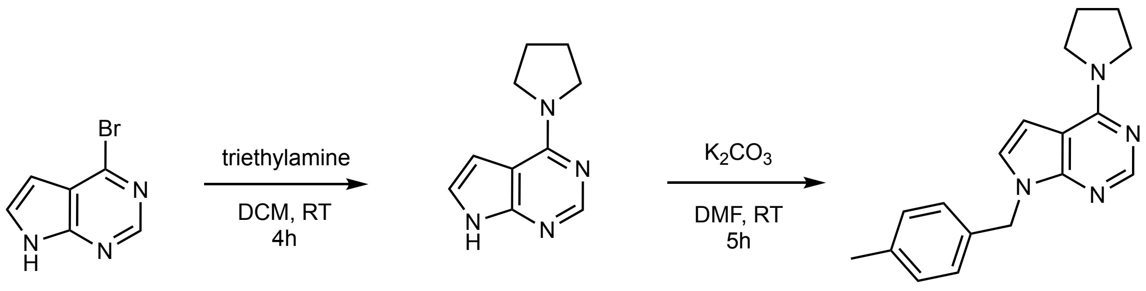

Intermediate B

4310-008
(BRD4310a)

To a mixture of 4-bromo-7H-pyrrolo[2,3-d]pyrimidine (5.00 g, 25.25 mmol, 1 eq) and TEA (triethylamine, 5.11 g, 50.50 mmol, 2 eq) in DCM (dichloromethane, 100 mL) was added pyrrolidine (2.15 g, 30.30 mmol, 1.2 eq). The resulting mixture was stirred at room temperature for 4 h. The reaction mixture was diluted with water (100 mL). The organic phase was separated and the aqueous phase was extracted with EA (ethyl acetate, 100 mL×3). The combined organic phase was washed with brine, dried over anhydrous Na_2SO_4 and concentrated in vacuo. The residue was purified by silica gel chromatography to give 4 g of **Intermediate B**. Yield was 84%. LC-MS m/z calc. for $C_{10}H_{12}N_4$ $[M+H]^+$ = 189.1, found 189.1.

To a mixture of **Intermediate B** (100 mg, 0.53 mmol, 1 eq) in DMF (dimethylformamide, 2.6 mL) were added 1-(chloromethyl)-4-methylbenzene (150 mg, 1.06 mmol, 2 eq) and K_2CO_3 (146 mg,

1.06 mmol, 2 eq). The resulting mixture was stirred at room temperature for 5 h. The reaction mixture was filtered and the filtrate was concentrated in vacuo. The residue was purified via prep-HPLC to provide **BRD4310a** (i.e., **4310-008**) in yield 41% (86 mg). LC-MS m/z calc. for $C_{18}H_{20}N_4$ $[M+H]^+$ 293.2, found: 293.1, t_R = 1.8 min. ^1H NMR (400 MHz, $CDCl_3$) δ 8.44 (s, J = 10.1 Hz, 1H), 7.13 (dd, J = 8.6, 2.7 Hz, 4H), 7.10 (d, J = 3.5 Hz, 1H), 6.72 (t, J = 6.9 Hz, 1H), 5.38 (s, 2H), 3.97 (s, 4H), 2.32 (s, 3H), 2.16 (s, 4H).

BRD4310a ^{1}H NMR and LC-MS:

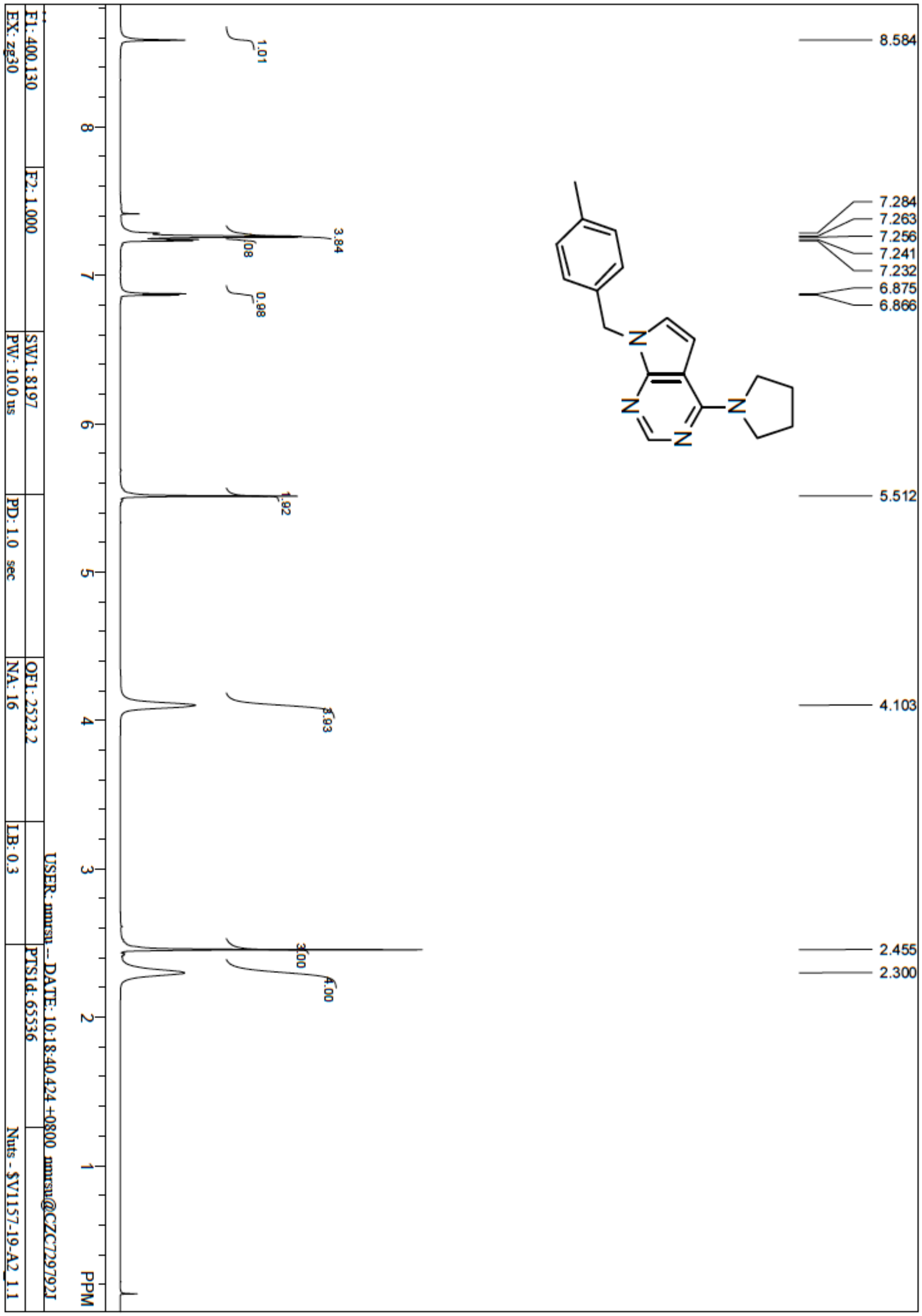

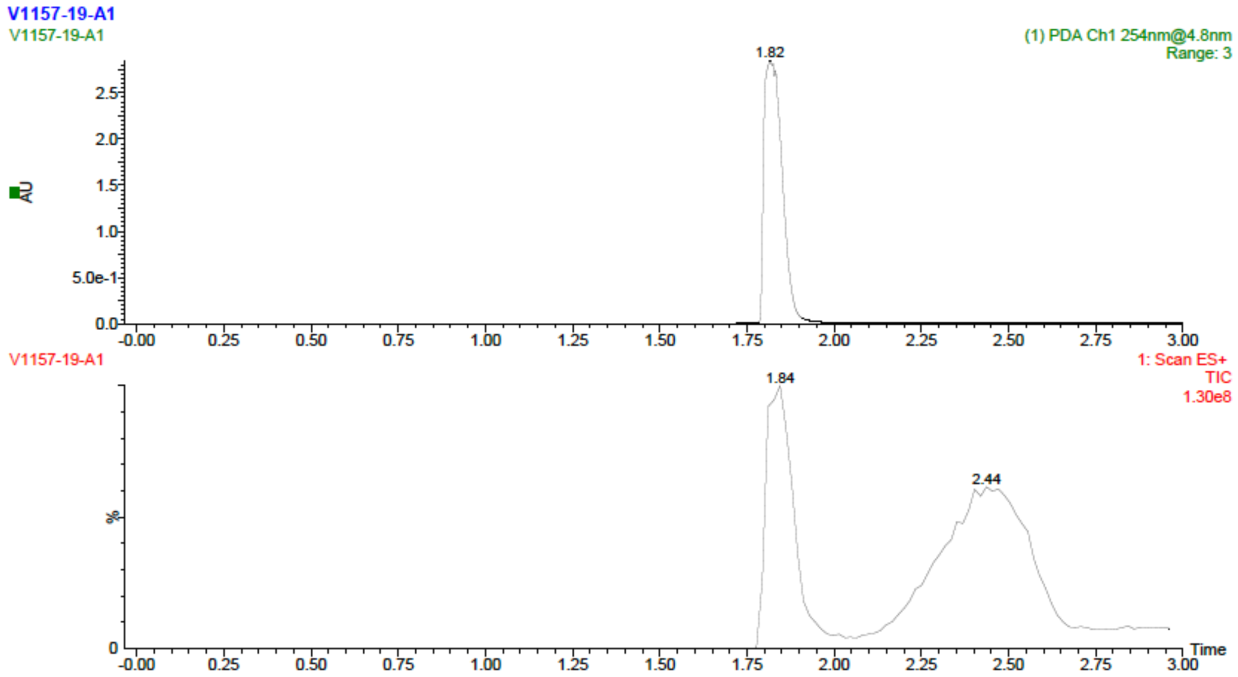
V1157-19-A1
V1157-19-A1
(1) PDA Ch1 254nm@4.8nm
Range: 3
1.82
AU
V1157-19-A1
1: Scan ES+
TIC
1.30e8
1.84
2.44
%
Time

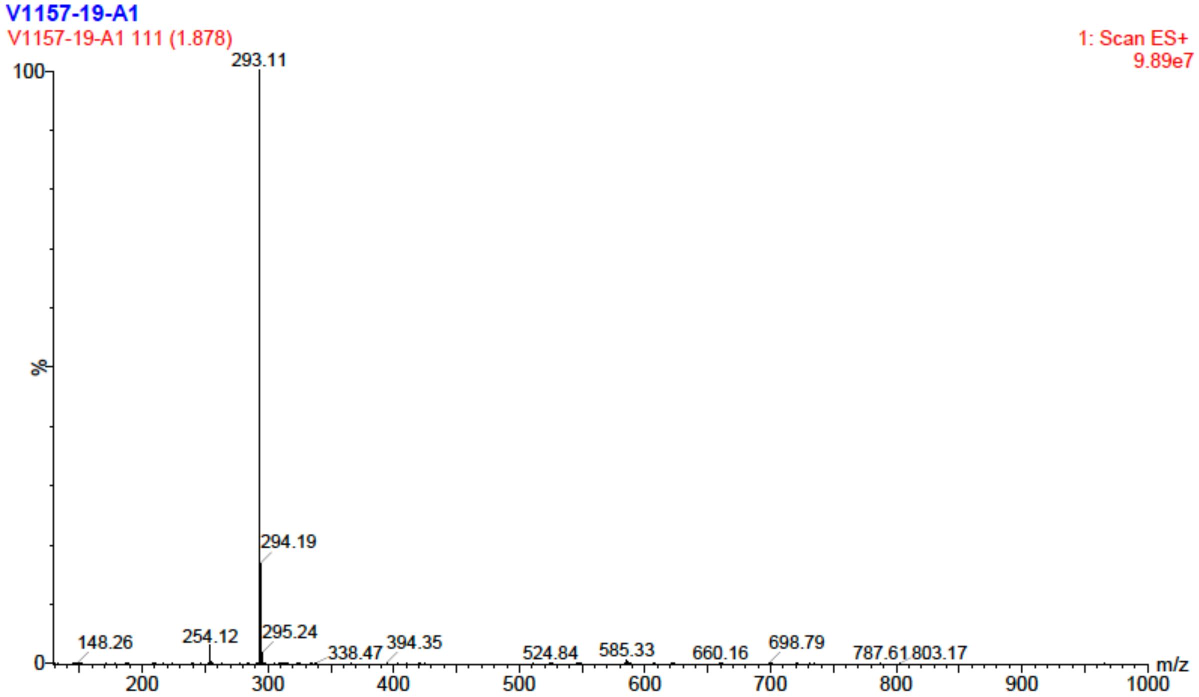
V1157-19-A1
V1157-19-A1 111 (1.878)
1: Scan ES+
9.89e7
293.11
294.19
295.24
148.26
254.12
338.47
394.35
524.84
585.33
660.16
698.79
787.61
803.17
%
m/z

REFERENCES

1 Johnson, E. O. *et al.* Large-scale chemical-genetics yields new *M. tuberculosis* inhibitor classes. *Nature* **571**, 72-78 (2019). https://doi.org/10.1038/s41586-019-1315-z

2 Enache, O. M. *et al.* The GCTx format and cmapPy, R, M, J packages: resources for optimized storage and integrated traversal of annotated dense matrices. *Bioinformatics* **35**, 1427-1429 (2019). https://doi.org/10.1093/bioinformatics/bty784

3 Hafner, M., Niepel, M., Chung, M. & Sorger, P. K. Growth rate inhibition metrics correct for confounders in measuring sensitivity to cancer drugs. *Nat Methods* **13**, 521-527 (2016). https://doi.org/10.1038/nmeth.3853

4 Bolstad, B. M., Irizarry, R. A., Astrand, M. & Speed, T. P. A comparison of normalization methods for high density oligonucleotide array data based on variance and bias. *Bioinformatics* **19**, 185-193 (2003). https://doi.org/10.1093/bioinformatics/19.2.185

5 Subramanian, A. *et al.* A Next Generation Connectivity Map: L1000 Platform and the First 1,000,000 Profiles. *Cell* **171**, 1437-1452 e1417 (2017). https://doi.org/10.1016/j.cell.2017.10.049

6 Ahlmann-Eltze, C. & Huber, W. Comparison of transformations for single-cell RNA-seq data. *Nat Methods* **20**, 665-672 (2023). https://doi.org/10.1038/s41592-023-01814-1

7 Durbin, B. P., Hardin, J. S., Hawkins, D. M. & Rocke, D. M. A variance-stabilizing transformation for gene-expression microarray data. *Bioinformatics* **18 Suppl 1**, S105-110 (2002). https://doi.org/10.1093/bioinformatics/18.suppl_1.s105

8 Eling, N., Richard, A. C., Richardson, S., Marioni, J. C. & Vallejos, C. A. Correcting the Mean-Variance Dependency for Differential Variability Testing Using Single-Cell RNA Sequencing Data. *Cell Syst* **7**, 284-294 e212 (2018). https://doi.org/10.1016/j.cels.2018.06.011

9 Love, M. I., Huber, W. & Anders, S. Moderated estimation of fold change and dispersion for RNA-seq data with DESeq2. *Genome Biol* **15**, 550 (2014). https://doi.org/10.1186/s13059-014-0550-8

10 Zhao, Y., Wong, L. & Goh, W. W. B. How to do quantile normalization correctly for gene expression data analyses. *Sci Rep* **10**, 15534 (2020). https://doi.org/10.1038/s41598-020-72664-6

11 Zwiener, I., Frisch, B. & Binder, H. Transforming RNA-Seq data to improve the performance of prognostic gene signatures. *PLoS One* **9**, e85150 (2014). https://doi.org/10.1371/journal.pone.0085150

12 Becht, E. *et al.* Dimensionality reduction for visualizing single-cell data using UMAP. *Nat Biotechnol* (2018). https://doi.org/10.1038/nbt.4314

13 Malyutina, A., Tang, J., & Pessia, A. . drda: An R Package for Dose-Response Data Analysis Using Logistic Functions. *Journal of Statistical Software* **106**, 1-26 (2023). https://doi.org/10.18637/jss.v106.i04

14 Akaike, H. A new look at the statistical model identification. *IEEE Transactions on Automatic Control* **19**, 716-723 (1974). https://doi.org/10.1109/TAC.1974.1100705

15 Ng, A., Jordan, M. & Weiss, Y. On Spectral Clustering: Analysis and an algorithm. *Adv. Neural Inf. Process. Syst* **14** (2002).

16 von Luxburg, U. A tutorial on spectral clustering. *Statistics and Computing* **17**, 395-416 (2007). https://doi.org/10.1007/s11222-007-9033-z

17 Arthur, D. V., S. in *SODA '07: Proceedings of the Eighteenth Annual ACM-SIAM Symposium on Discrete Algorithms.* 1027–1035.
18 Lloyd, S. Least Squares Quantization in PCM. *IEEE Transactions on Information Theory* **28**, 129-137 (1982).
19 Aly, M. Survey on multiclass classification methods. (California Institute of Technology, Caltech, 2005).
20 Bishop, C. M. *Pattern recognition and machine learning*. (Springer, 2006).
21 Yeang, C. H. *et al.* Molecular classification of multiple tumor types. *Bioinformatics* **17 Suppl 1**, S316-322 (2001). https://doi.org/10.1093/bioinformatics/17.suppl_1.s316
22 Molinaro, A. M., Simon, R. & Pfeiffer, R. M. Prediction error estimation: a comparison of resampling methods. *Bioinformatics* **21**, 3301-3307 (2005). https://doi.org/10.1093/bioinformatics/bti499
23 Robin, X. *et al.* pROC: an open-source package for R and S+ to analyze and compare ROC curves. *BMC Bioinformatics* **12**, 77 (2011). https://doi.org/10.1186/1471-2105-12-77
24 Maggiora, G., Vogt, M., Stumpfe, D. & Bajorath, J. Molecular similarity in medicinal chemistry. *J Med Chem* **57**, 3186-3204 (2014). https://doi.org/10.1021/jm401411z
