# Supplementary material for: Reference-based chemical-genetic interaction profiling to elucidate small molecule mechanism of action in Mycobacterium tuberculosis
Source: Nat Commun. 2025 Nov 3;16:9673. doi: 10.1038/s41467-025-64662-x (PMC12583738; doi:10.1038/s41467-025-64662-x)
Supplement: Supplementary file 2 — Description of Additional Supplementary Files [file 41467_2025_64662_MOESM2_ESM.pdf]

Title: Supplementary Data 1

Description: Reference set of compounds with annotated mechanism of action (MOA) used for constructing Perturbagen Class (PCL) clusters and making reference-based MOA predictions.

Title: Supplementary Data 2

Description: Strains used in chemical genetic screens and their average baseline growth rates across the PROSPECT screening waves.

Title: Supplementary Data 3

Description: Reference set chemical-genetic interaction (CGI) profiles belonging to Perturbagen Class (PCL) clusters and the PCL cluster sizes.

Title: Supplementary Data 4

Description: Reference-based mechanism of action (MOA) predictions for reference set compounds in leave-one-out cross-validation (LOOCV) using Perturbagen Class (PCL) analysis.

Title: Supplementary Data 5

Description: Reference-based mechanism of action (MOA) predictions for GlaxoSmithKline (GSK) set compounds using Perturbagen Class (PCL) analysis.

Title: Supplementary Data 6

Description: Maximum Tanimoto similarities between GlaxoSmithKline (GSK) set and BRD4310 and reference set compounds.
