# Supplementary material for: Reference-based chemical-genetic interaction profiling to elucidate small molecule mechanism of action in Mycobacterium tuberculosis
Source: Nat Commun. 2025 Nov 3;16:9673. doi: 10.1038/s41467-025-64662-x (PMC12583738; doi:10.1038/s41467-025-64662-x)
Supplement: Supplementary file 9 — Reporting Summary [file 41467_2025_64662_MOESM9_ESM.pdf]

nature portfolio

Corresponding author(s): Deborah T. Hung
James E. Gomez

Last updated by author(s): Sep 15, 2025

Reporting Summary

Nature Portfolio wishes to improve the reproducibility of the work that we publish. This form provides structure for consistency and transparency in reporting. For further information on Nature Portfolio policies, see our Editorial Policies and the Editorial Policy Checklist.

Statistics

For all statistical analyses, confirm that the following items are present in the figure legend, table legend, main text, or Methods section.

n/a	Confirmed	
☐	☒	The exact sample size (*n*) for each experimental group/condition, given as a discrete number and unit of measurement
☐	☒	A statement on whether measurements were taken from distinct samples or whether the same sample was measured repeatedly
☐	☒	The statistical test(s) used AND whether they are one- or two-sided<br>*Only common tests should be described solely by name; describe more complex techniques in the Methods section.*
☐	☒	A description of all covariates tested
☐	☒	A description of any assumptions or corrections, such as tests of normality and adjustment for multiple comparisons
☐	☒	A full description of the statistical parameters including central tendency (e.g. means) or other basic estimates (e.g. regression coefficient) AND variation (e.g. standard deviation) or associated estimates of uncertainty (e.g. confidence intervals)
☐	☒	For null hypothesis testing, the test statistic (e.g. *F*, *t*, *r*) with confidence intervals, effect sizes, degrees of freedom and *P* value noted<br>*Give P values as exact values whenever suitable.*
☒	☐	For Bayesian analysis, information on the choice of priors and Markov chain Monte Carlo settings
☒	☐	For hierarchical and complex designs, identification of the appropriate level for tests and full reporting of outcomes
☐	☒	Estimates of effect sizes (e.g. Cohen's *d*, Pearson's *r*), indicating how they were calculated

Our web collection on statistics for biologists contains articles on many of the points above.

Software and code

Policy information about availability of computer code

Data collection	For processing raw sequence data from Illumina, PicardTools and Samtools. Fluorescence, luminescence, and absorbance plate reader data was collected using Molecular Devices SoftMax Pro

Data analysis	GraphPad Prism versions 10.2.3 was used for data analysis and graphing for MIC, resistance mutants studies, and ATP assay.<br>Data were archived and analyzed using the CDD Vault from Collaborative Drug Discovery (Burlingame, CA. www.collaborativedrug.com)<br>Microsoft Excel 16.89.1<br>FastQC (version 0.11.5) and DESeq2 (version 1.28.1) were used for RNA-seq data analysis.<br>R versions 4.1, 4.4 including cmapR (version 1.6.0), umap (version 0.2.7.0), drda (version 2.0.2), tidyverse (version 1.3.1), and ggpubr (version 0.6.0)<br>Matlab versions R2020a, R2020b including Bioinformatics Toolbox, Parallel Computing Toolbox, and Statistics and Machine Learning Toolbox<br>Python version 3.7.12 including RDKit (version 2020.09.1)<br>For nucleotide sequence analysis, Samtools, GATK, Pilon<br>ConcensusGLM is available at http://github.com/eachanjohnson/concensusGLM<br>CmapM is available on GitHub at https://github.com/cmap/cmapM<br>CmapR is available through Bioconductor and on GitHub at https://github.com/cmap/cmapR<br>Computer code for running each step of the reference-based PCL analysis is available on Code Ocean at https://doi.org/10.24433/CO.3013890.v1 and GitHub at https://github.com/broadinstitute/Mtb_PROSPECT_PCL_analysis<br>Other custom computer code is available from the corresponding author upon request<br>Heatmap visualization was performed in Morpheus at https://software.broadinstitute.org/morpheus

For manuscripts utilizing custom algorithms or software that are central to the research but not yet described in published literature, software must be made available to editors and reviewers. We strongly encourage code deposition in a community repository (e.g. GitHub). See the Nature Portfolio guidelines for submitting code & software for further information.

Data

Policy information about availability of data

All manuscripts must include a data availability statement. This statement should provide the following information, where applicable:

- Accession codes, unique identifiers, or web links for publicly available datasets
- A description of any restrictions on data availability
- For clinical datasets or third party data, please ensure that the statement adheres to our policy

Source data are provided with this paper. The standardized growth rate (sGR), Pearson correlation to reference CGI profiles, average rank of Pearson correlation across reference CGI profiles, reference CGI profile PCL cluster membership, PCL similarity score, and PCL confidence score data for reference CGI profiles, the GSK set, and BRD4310 are available online on Code Ocean within the published code capsule at https://doi.org/10.24433/CO.3013890.v1 and have been deposited in Figshare: https://doi.org/10.6084/m9.figshare.28373561. The reference set and GSK compound MOA annotations are available within Supplementary Data 1 and Supplementary Data 5, respectively. RNA-seq and resistant mutant whole-genome sequencing data have been deposited in the NCBI Sequence Read Archive under accession code PRJNA1328039.

Research involving human participants, their data, or biological material

Policy information about studies with human participants or human data. See also policy information about sex, gender (identity/presentation), and sexual orientation and race, ethnicity and racism.

Reporting on sex and gender	not applicable
Reporting on race, ethnicity, or other socially relevant groupings	not applicable
Population characteristics	not applicable
Recruitment	not applicable
Ethics oversight	not applicable

Note that full information on the approval of the study protocol must also be provided in the manuscript.

Field-specific reporting

Please select the one below that is the best fit for your research. If you are not sure, read the appropriate sections before making your selection.

☒ Life sciences ☐ Behavioural & social sciences ☐ Ecological, evolutionary & environmental sciences

For a reference copy of the document with all sections, see nature.com/documents/nr-reporting-summary-flat.pdf

Life sciences study design

All studies must disclose on these points even when the disclosure is negative.

Sample size	No statistical methods were used to predetermine sample size. Sample sizes (n = 2) for screening were chosen as standard for high-throughput compound screening as a balance of cost and accuracy. The PROSPECT screening assay was previously optimized to allow 2 replicates to provide statistical power as described in Johnson et al. 2019. https://doi.org/10.1038/s41586-019-1315-z. All results from the screening were confirmed by orthogonal methods, whose sample size was chosen (n = 2-3) from experience to provide power and accuracy.

Data exclusions	For each screening wave independently, strains that grew slowly (did not achieve at least one doubling over the 14-day assay) or unreliably (any conditions with GR > 20) were identified and filtered out from further analysis. Of the 387 strains that were included in the strain pool across all six screening waves, 42 strains were filtered out for slow growth in at least one of the screening waves and 5 strains were filtered out for high GR; the 340 strains that passed quality control in all six screening waves were used in downstream analysis. Otherwise, no data were excluded from the analyses presented.
Replication	Primary data were generated in at least duplicate and were shown to give similar results. Results were confirmed using orthogonal methods, which demonstrated the reliability of the primary data as described in the manuscript. Follow-up mechanism of action studies were replicated at least 3 times. Due to limited compound availability, testing of GSK compounds against cydA::Tn and qcrB mutants was performed in duplicate. All attempts at replication were successful.
Randomization	The experiments were not randomized. Plate-to-plate and well-to-well variation in screening was accounted for by using well spike-in control barcode counts to log2-normalize for PCR and sequencing coverage differences as described in the manuscript and through modeling covariate effects (i.e., batch effects) with a generalized linear model using ConcensusGLM as previously described and cited in the manuscript.
Blinding	Investigators were not blinded during data collection or analysis for reasons of feasibility. Compounds in our study were assigned ID numbers, essentially blinding their identity until after collection and analysis was complete. MOA predictions for the GSK set compounds were performed blinded to their chemical identity or other associated information. Follow-up communication with GSK unblinded the chemical identities of the compounds and allowed for literature review of reported mechanistic activities. Follow-up mechanism of action studies were not blinded due to experimental feasibility.

Reporting for specific materials, systems and methods

We require information from authors about some types of materials, experimental systems and methods used in many studies. Here, indicate whether each material, system or method listed is relevant to your study. If you are not sure if a list item applies to your research, read the appropriate section before selecting a response.

Materials & experimental systems

n/a	Involved in the study
☒	☐ Antibodies
☒	☐ Eukaryotic cell lines
☒	☐ Palaeontology and archaeology
☒	☐ Animals and other organisms
☒	☐ Clinical data
☒	☐ Dual use research of concern
☒	☐ Plants

Methods

n/a	Involved in the study
☒	☐ ChIP-seq
☒	☐ Flow cytometry
☒	☐ MRI-based neuroimaging

Plants

Seed stocks	*Report on the source of all seed stocks or other plant material used. If applicable, state the seed stock centre and catalogue number. If plant specimens were collected from the field, describe the collection location, date and sampling procedures.*
Novel plant genotypes	*Describe the methods by which all novel plant genotypes were produced. This includes those generated by transgenic approaches, gene editing, chemical/radiation-based mutagenesis and hybridization. For transgenic lines, describe the transformation method, the number of independent lines analyzed and the generation upon which experiments were performed. For gene-edited lines, describe the editor used, the endogenous sequence targeted for editing, the targeting guide RNA sequence (if applicable) and how the editor was applied.*
Authentication	*Describe any authentication procedures for each seed stock used or novel genotype generated. Describe any experiments used to assess the effect of a mutation and, where applicable, how potential secondary effects (e.g. second site T-DNA insertions, mosiacism, off-target gene editing) were examined.*
